# From hogs to HABs: impacts of industrial farming in the US on nitrogen and phosphorus and greenhouse gas pollution

**DOI:** 10.1007/s10533-020-00691-6

**Published:** 2020-08-10

**Authors:** Patricia M. Glibert

**Affiliations:** Horn Point Laboratory, University of Maryland Center for Environmental Science, PO Box 775, Cambridge, MD 21613 USA

**Keywords:** N and P fertilizer, Manure, Factory farms, CAFOs, Eutrophication, Algal blooms, Atmospheric deposition, Greenhouse gasses

## Abstract

Nutrient pollution and greenhouse gas emissions related to crop agriculture and confined animal feeding operations (CAFOs) in the US have changed substantially in recent years, in amounts and forms. This review is intended to provide a broad view of how nutrient inputs—from fertilizer and CAFOs—as well as atmospheric NH_3_ and greenhouse gas emissions, are changing regionally within the US and how these changes compare with nutrient inputs from human wastewater. Use of commercial nitrogen (N) fertilizer in the US, which now exceeds 12,000,000 metric tonnes (MT) continues to increase, at a rate of 60,000 MT per year, while that of phosphorus (P) has remained nearly constant over the past decade at around 1,800,000 MT. The number of CAFOs in the US has increased nearly 10% since 2012, driven largely by a near 13% increase in hog production. The annualized inventory of cattle, dairy cows, hogs, broiler chickens and turkeys is approximately 8.7 billion, but CAFOs are highly regionally concentrated by animal sector. Country-wide, N applied by fertilizer is about threefold greater than manure N inputs, but for P these inputs are more comparable. Total manure inputs now exceed 4,000,000 MT as N and 1,400,000 MT as P. For both N and P, inputs and proportions vary widely by US region. The waste from hog and dairy operations is mainly held in open lagoons that contribute to NH_3_ and greenhouse gas (as CH_4_ and N_2_O) emissions. Emissions of NH_3_ from animal waste in 2019 were estimated at > 4,500,000 MT. Emissions of CH_4_ from manure management increased 66% from 1990 to 2017 (that from dairy increased 134%, cattle 9.6%, hogs 29% and poultry 3%), while those of N_2_O increased 34% over the same time period (dairy 15%, cattle 46%, hogs 58%, and poultry 14%). Waste from CAFOs contribute substantially to nutrient pollution when spread on fields, often at higher N and P application rates than those of commercial fertilizer. Managing the runoff associated with fertilizer use has improved with best management practices, but reducing the growing waste from CAFO operations is essential if eutrophication and its effects on fresh and marine waters–namely hypoxia and harmful algal blooms (HABs)—are to be reduced.

**Electronic supplementary material:**

The online version of this article (10.1007/s10533-020-00691-6) contains supplementary material, which is available to authorized users.

## Introduction

In the 1970s, eutrophication from nitrogen (N) and phosphorus (P) pollution was a problem largely localized to some freshwaters (e.g., Likens 1972, Ketchum 1972), and the major source of nutrient pollution was considered to be sewage wastewater. At that time the US population was about 200 million, but by 2019, population had increased to 328 million (https://www.multpl.com/united-states-population/table/by-year). Eutrophication is the cause of hypoxia zones that have now been documented in most US estuaries and along many coasts (e.g., Cloern 2001; Howarth et al. 2002, Bricker et al. 2007 and references therein) and such zones are increasing worldwide (Diaz and Rosenberg 2008, Kemp et al. 2009; Rabalais et al. 2009, 2010). Freshwater eutrophication is an equally serious US and global problem (e.g., Smith et al. 2006; Du et al. 2019). The corn-belt of the US, the massive 39 million-ha span (primarily encompassing the states of Illinois, Indiana, Iowa, Missouri and Ohio) that uses more than 4.5 million metric tonnes (MT) of chemical N fertilizer and nearly a million MT of N from manure for the growth of corn and soybean (Foley 2013), is considered to be the source of the N fueling the dead zone in the Gulf of Mexico, one of the largest hypoxic zones in the US (e.g., Scavia et al. 2003; Turner et al. 2006; Alexander et al. 2008). Eutrophication is also highly correlated with the increasing frequency and geographic spread of both freshwater and coastal marine harmful algal blooms (HABs; Anderson et al. 2002; Heisler et al. 2008; Glibert et al. 2005, 2014, 2018). These events have now been documented in every state, and recent examples of algal blooms affecting drinking water (Anderson et al. 2008; Steffen et al. 2017), fisheries closures and human health issues are regularly reported throughout the country (e.g., Fleming et al. 2005; Backer et al. 2005; Backer and McGillicuddy 2006; McCabe et al. 2016 among others). Throughout the world, excess N and P have led to a cascade of atmospheric, water and human health problems and managing nutrient pollution has become a grand challenge (e.g., Galloway et al. 2003; Townsend et al. 2003; Howarth 2008; Billen et al. 2013; Sutton et al. 2013; Davidson et al. 2015; Glibert et al. 2014, 2018; Glibert and Burford 2017; Glibert 2020).

In the 1970s, greenhouse gases were only just beginning to be recognized as a threat to future global warming. Since then, global greenhouse gas emissions have increased 75%, with a 25% increase from the 1990s to 2004 alone, primarily due to increases in fossil fuel use globally, but particularly from the rapid industrial development in China and other developing countries (https://www.pbl.nl/en/dossiers/Climatechange/TrendGHGemissions1990-2004). However, agriculture also contributes to this increase, such that by 2017, agricultural sources contributed 10–15% of greenhouse gas emissions in the US (https://www.epa.gov/ghgemissions/sources-greenhouse-gas-emissions; Grossi et al. 2019). Agriculture contributes to such emissions in multiple ways, including direct emissions from livestock (enteric fermentation), and as will be shown below, from handling of animal waste and from fertilizer applications.

Although agriculture-related eutrophication problems have escalated in the past few decades, farming practices actually began to change rapidly after World War II. The so-called Green Revolution, the period during which the manufacture and application of N-based fertilizers expanded at a rapid pace also included other advances in farming technology, such as improved irrigation, mechanized equipment and better seeds (e.g., Smil 2001; Erisman et al. 2008; Pingali 2012). As described by Imhoff (2019, p. 33), “Chemicals were concocted into a slew of pesticides, herbicides and synthetic fertilizers… Plant breeding also evolved, creating high-yielding hybrid grains tailored to meet these shifts in chemical inputs and mechanical growing and harvesting”. Thus, compared to pre-industrial times, the US has seen a > fivefold increase in N use on average, but this increase has been up to > 35-fold in some regions of the country (Houlton et al. 2013; Sobota et al. 2015).

Increased fertilizer use led to rising grain yields, but also an oversupply of grains. The US did not become the world’s breadbasket by grand or moral intentions, but rather because, as farming became more intensive, there was a surplus and a need to find new markets for products and a desire to raise domestic profits (Walker 2019). The US consequently adopted policies that have promoted the “feeding of the world” in order to sustain profitability (e.g., Imhoff 2019). The US now produces a total weight in corn that is, “remarkably close to the estimated weight of the global population,” about 287 million MT (Gunderson et al. 2018). By 2011, about a third of all US crops were exported (Hertel 2018).

Oversaturation of the market at various times has also led to further plowing of the ground for more crops to make up for lost income. The motive is to grow the most high-yielding, high-paying crop.

The US Farm Bill, the major legislation that encompasses agriculture, conservation, and research and food assistance programs, has, over its various iterations and re-authorizations, incentivized monoculture production, primarily corn and soybean. Its major objective is to stabilize prices and incomes, not to protect environmental interests (Ruhl 2000). This massively expensive legislation guides all aspects of the US food and farming systems, but is heavily influenced by special interests, and thus its policies have favored consolidated large-scale farms, and grains over fruits and vegetables, heavy use of chemical fertilizers, among other incentives to maximize profits over environmental stewardship (e.g., Miller 2017; Imhoff 2019).

Because of these shifts and other policy- or economic-related factors, most of the grain grown in US is not used directly for food. It is fed to animals in feedlots (about 36%), used for biofuels (about 40%), exported (about 10%), and used in high-fructose corn syrup and other food products (a few %; Foley 2013; Barton and Clark 2014). Of the total acreage in corn, about 5%, or 2 million ha, is needed just to support the supply of chicken and pork sold at McDonald’s and Walmart (von Reusner 2019). Only ~ 1% of all corn grown is directly eaten by people as “sweet corn” (Bittman 2019). The mandate for ethanol production in the US, originally intended to support farmers and reduce foreign dependence on oil, has resulted in 12.5 million ha of corn dedicated to ethanol corn (equivalent to more than all the crop land in Iowa; Imhoff 2019) and likely has contributed to an increase in N fertilizer use in the past 2 decades (e.g., Sabo et al. 2019). In the 1990s, the US produced about 10 million MT of corn for biofuels; in 2018 it was ~ 140 million MT, about 12-fold more than that used for high fructose corn syrup (https://www.worldofcorn.com/#us-corn-at-a-glance). Recent trade tariffs notwithstanding, this demand will continue.

The factory-efficient approach to farming has gone hand-in-hand with changing diets (e.g., Godfrey et al. 2018). People consume more protein—as meat—when wealth increases and as the cost of meat production decreases. Cattle, otherwise adapted to grass, are fed corn because it is a cheap commodity, because “the great pile must be consumed”, and because animals can grow to market size much more quickly (Pollan 2006, p. 68). Notable, however, is the fact that the nutritional content of corn-fed beef differs from that of grass-fed beef, with more saturated fat and less omega-3-fatty acids (Pollan 2006). Similarly, corn-fed chickens grow much faster and larger than free-range chickens. Broiler chickens are now about 12% larger than those grown just a decade ago (Pelton et al. 2020).

**Fig. 1:**
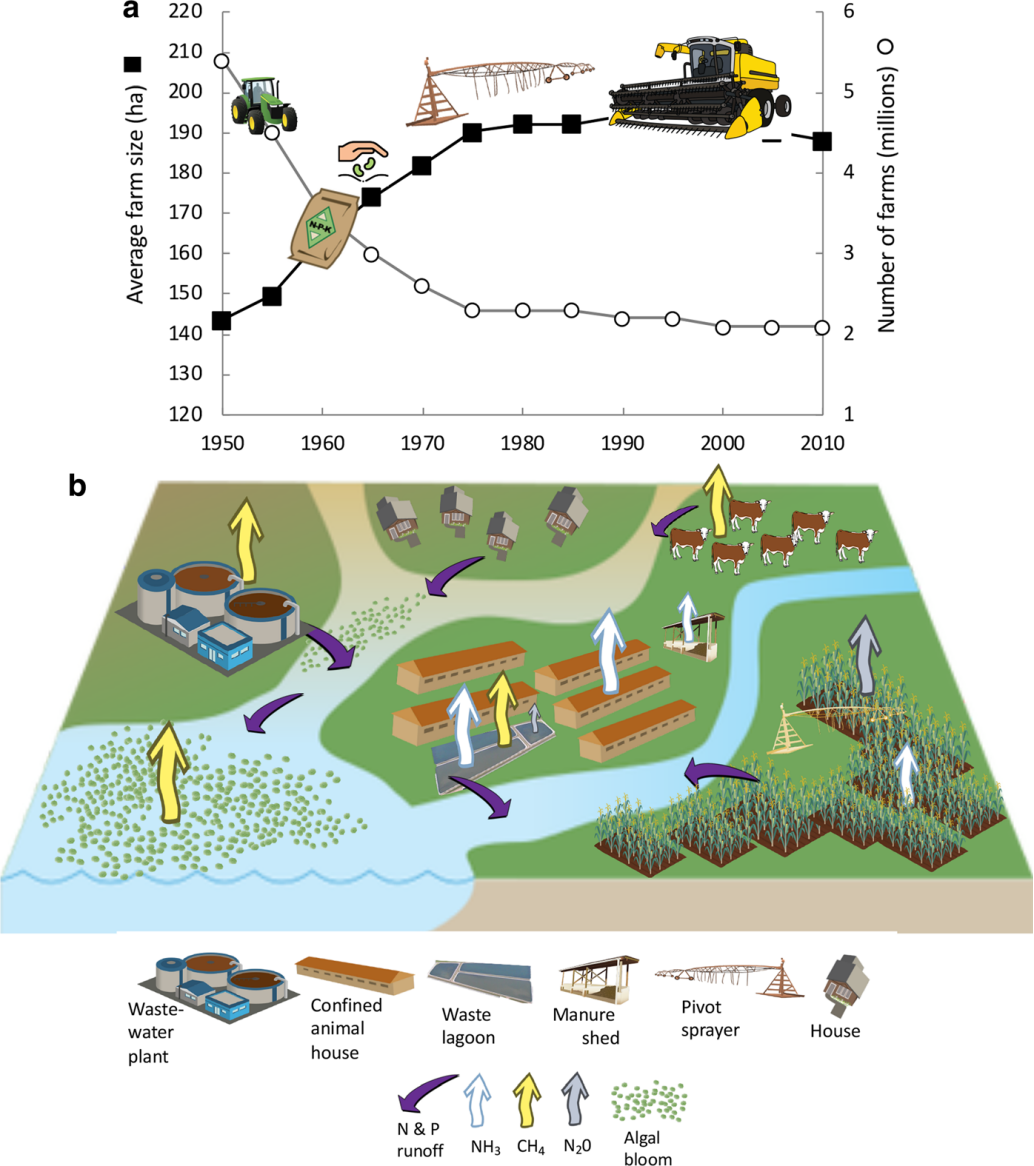
**a** Change in the average US farm size and number of farms with time. **b** Conceptual schematic of the sources of nitrogen and phosphorus runoff and ammonia and greenhouse gas emissions and effects on algal blooms considered herein. Symbols and icons are from the University of Maryland Center for Environmental Science (UMCES) Integration and Application Network (IAN) image library or from Vectorstock used under an expanded license

Concentrated animal feeding operations (CAFOs) began increasing rapidly in the 1990s (e.g., Mallin 2000) as the most economically efficient way to produce the quantity of meat needed. The number of animals per farm and the scale and size of farms increased, while the number of farms decreased; small animal farms were simply no longer economically viable (Fig. 1a). Accordingly, “In one generation, the number of farms producing hogs fell by almost three quarters—while the median number of hogs per farm climbed from 1200 to 40,000” (Walker 2019, p. 35). Furthermore, agribusinesses have concentrated all aspects of animal production by buying companies in the same line of production *and* buying companies that had previously provided them with raw materials or sold finished products, such as meat packing plants. As noted by Walker (2019 p. 134, quoting journalist Barry Lynn), “If antitrust law exists to serve the consumer, and if consumers are best served by getting more for less, and if the best way to get more for less is to encourage business to be ‘efficient’, and if the best way to be efficient is to build up scale and scope, then ergo, monopoly is the best friend of the consumer”.

The proliferation of CAFOs is also a function of the aforementioned growth in corn and soybean production, as the over-production of these commodities depressed the price of livestock feed, which, in turn, created an indirect subsidy for animal production systems (Pollan 2006; Food and Water Watch 2015). Cheap animal feed translates into cheaper meat products. Packing large numbers of animals in confined spaces was also facilitated by the massive use of antibiotics (Walker 2019). In all, US farms, owned increasingly by a comparatively small number of companies, have become “too big to fail” (Walker 2019). Mega-farms owners can also buffer economic downturns far better than family farms.

The dietary change to increased consumption of meat is not just a US phenomenon; Chinese consumption of pork, poultry and beef has also increased and meat has become a more consistent dietary component as its economy has grown. China’s meat production, in fact, rose 250% from 1986 to 2012, with another 30% rise by 2020, and their need for animal feed is one of the major drivers of their escalation in importation of US and Brazilian soybeans over the past decade (Sheldon 2019). In China, farms with > 1000 head of cattle grew 16% from 2011 to 2014, while those of dairy cows grew 40%. A single Chinese dairy farm with > 100,000 head is currently being developed (DuBois and Gao 2017).

The numbers of animals in CAFOs differs widely, depending on the animal and regional permitting. CAFOs are categorized as small, medium, or large depending on the number and type of animal and the drainage system for their waste (Table 1). Small CAFOs (those with small animal populations just under the definition of medium-sized) are often undercounted or un-permitted and are expanding in many regions where regulations apply only to larger facilities. By keeping animal operations to numbers that do not fall into the category for regulation, operators maintain more options—and more polluting options—for handling waste. Current permitting and legal differences between states makes it difficult to obtain an accurate count of the number of CAFOs in the US. Transparency of CAFO data, with respect to permit state, location, manure storage or type, and number of animals is low for almost every state; the US Environmental Protection Agency (US EPA) does not have such data for about half of the CAFOs in its inventory of 2012 (Miller and Muren 2019). New algorithms are being applied to obtain better estimates and these approaches suggest that the number of CAFOs is actually more than 15% higher that which is routinely reported from manual enumerations (Handan-Nader and Ho 2019). Thus, the numbers reported herein are likely similarly underestimated.

**Table 1:** Definitions of large and medium CAFOs according to USEPA (https://www3.epa.gov/npdes/pubs/sector_table.pdf)

Animal type	Large	Medium*
Cattle	> 1000	300–999
Dairy	> 700	200–699
Swine (> 55 lbs)	> 2500	750–2499
Swine (< 55 lbs)	> 10,000	3000–9909
Broilers	> 125,000	37,500–124,999
Layers	> 82,000	25,000–81,999

Note that there are many animals in confined conditions in operations with numbers fewer than indicated here and thus are undercounted in this analysis. Small CAFOS have numbers of animals less than those defined for “medium”

*Medium either has animals in range above or has a manmade ditch or pipe that carries manure or wastewater to surface water or the animals come into contact with surface water that passes through the area where they are confined

Given the density of animals in CAFOs, and the rate at which animals are fed to get them to market as quickly as possible, the amount of animal waste from these operations can be very large (e.g., Cahoon et al. 1999; Mallin 2000, Mallin and Cahoon 2003, Burkholder et al. 2007, Mallin et al. 2015). Although the waste produced by CAFOs across the US is examined in this review, as an example of the scale of this nutrient source, in the Cape Fear River basin of North Carolina, it was estimated that in the early 2000s, there were 5 million hogs, 300 million chickens, and 16 million turkeys produced annually on ~ 2000 CAFOs, yielding 82,700 MT of N and 26,000 MT of P (Mallin et al. 2015 and references therein). Moreover, in the Chesapeake Bay region, where poultry production has increased 6% in the past decade, the manure production from these CAFOs has actually increased 16% because larger, more meaty chickens are being grown (Pelton et al. 2020).

Collectively, farming practices today contribute substantially to N and P pollution of waterways and to NH_3_ and greenhouse gas emissions (Fig. 1b). Most CAFOs produce waste at a scale that is more than can be accommodated by the method by which manure was traditionally handled, that is, by spreading it on adjacent land as fertilizer (as dry litter for poultry and as liquid manure for hog and dairy manure; Mallin et al. 2015). There is no wastewater treatment for these animal wastes—other than holding it for periods of time. While much is spread on land, most of the waste from dairy or hog operations is held in large, open-pit lagoons. The breeching of these lagoons during flooding and hurricanes has been a major pollution problem for states such as North Carolina with their large hog population. Many of North Carolina’s CAFOs are built on flood plains (www.ecowatch.com/factory-farm-waste-north-carolina-2628852719.htm) where land is comparatively inexpensive (but note that a moratorium has been in place since 1997 disallowing any new lagoons to be constructed in North Carolina). Following Hurricanes Florence in 2018, 33 such lagoons overflowed, spilling over 30 trillion L of wastes, together with thousands of dead hogs, repeating events of years earlier when Hurricane Floyd in 1999 led to spillage of 9 trillion L of hog waste (Buford 2018). In addition to the waste that makes its way into waterways, the volatilization of animal wastes and manures contributes to atmospheric deposition of NH_3_/NH_4_^+^, which has been shown to account for approximately half of the atmospheric N deposition in Mid-Atlantic estuaries such as the Neuse River Estuary and Atlantic coastal waters (Paerl, 1997; Whitall et al., 2003). Each broiler chicken, for example, emits between 0.27 and 0.54 g NH_3_ from its manure per day (Russ and Schaeffer 2018). Furthermore, and as will be described herein, liquid manure systems also contribute directly to greenhouse emissions, as CH_4_ and N_2_O.

The goal of this paper is to highlight inputs of nutrients and greenhouse gas pollution from farms in the US, by source, form, and by region of the country and their rapid changes over the recent years. There have been a number of recent inventories of fertilizer, manure and/or greenhouse gases in the US, built on modeling of a comprehensive suite of sources and fates (e.g., Ruddy et al. 2006; Sobota et al. 2015; Houlton et al. 2013; Swaney et al. 2018a, b; Bouwman et al. 2017; Sabo et al. 2019). Those efforts have focused on defining patterns and trends at fine spatial scales, i.e. at the level of counties or hydrologic units, and quantifying surpluses, not just sources. In contrast, this review is intended to provide the “30,000 ft” view of how nutrient inputs, from fertilizer and CAFOs, as well as atmospheric NH_3_ and greenhouse gas emissions, are changing regionally within the US and how these changes compare with nutrient inputs from human wastewater. By highlighting the rapid pace of changes in these important sources of environmental nutrient loads and other pollutants, these data may help to guide broad priorities for management actions for reduction of both water and air pollutants from these industrial operations; regional managers setting local nutrient reduction targets or strategies will want to consult the more detailed nutrient inventories. Although this paper specifically focuses on the US, there are important lessons that are applicable globally.

## Methods

### Overview

This paper begins with a review of the trends in total farms and their size. The change in use and form of chemical fertilizers (both N and P) in the US over time is then summarized as totals and for the major crops of corn, soybean, wheat, and cotton. The growth in major animal operations (including beef cattle, dairy, hogs, chickens as “broilers”, and turkeys) is then considered, as is the total numbers of CAFOs and their change regionally, and the total N and P released by animal type regionally. Emissions of NH_3_ and greenhouse gasses are then summarized. The N and P in human wastewater was estimated by state, along with overarching status of wastewater infrastructure by state. Data for these different sources of N and P were compared by aggregated US regions. Every effort was made to capture data from similar time periods for the different parameters; dates encompassed by the different trends are noted throughout.

### Data sources and calculations

Publicly-available and/or published data were accessed for all aspects of this analysis, and data sources are identified for each set of data used. Where assumptions or calculations were applied to available data, they are explicitly stated. Rates of change were calculated across various time periods depending on parameter and data availability.

The number and sizes of farms was obtained from https://cropinsuranceinamerica.org/in-the-states/ based on the year 2012. Data for 2017 were obtained from US Farm Data (www.usfarmdata.com/percentage-of-small-medium-and-large-farms-in-the-us).

Annual fertilizer statistics were obtained from the US EPA (https://www.epa.gov/nutrient-policy-data/commercial-fertilizer-purchased). These data are reported by crop and nutrient form. Data reported as P_2_O_5_ were herein converted to P using the factor 0.436. The US Department of Agriculture (USDA) have made available the total amount of N and P used by state in recent years (https://www.ers.usda.gov/data-products/fertilizer-use-and-price.aspx). Fertilizer data are based on available data through 2014; individual years are identified in comparative analyses. Other fertilizer data were obtained from the analyses of Sabo et al. (2019) for N and from comparable US EPA analyses for P (10.23719/1504278). These latter data, which are reported for 2002, 2007, and 2012, catalogued inputs and fates at the level of hydrologic units, roughly equivalent to medium-river-sized basins (HUC-8). These data were herein sorted and summed by state and then aggregated by US region.

Water use data by crop were from USDA (2008 as reported in Barton and Clark 2014).

Animal inventories were obtained from USDA (for 2012 from www.usda.gov/Publications/AgCensus/2012/Full-Report/Volume_1_Chapter_2_US_State_Level/; for 2016 and 2017 from www.aphis.usda/gov/animal-health/nahms/downloads/Demographics2017.pdf; and for 2019 from www.nass.usda.gov/Statistics_by_State/index.php). Animal inventory comparisons are herein focused on cattle, dairy cows, hogs, broiler chickens and turkeys, and while other animals may be inventoried and reported, these represent the major animals in polluting CAFO operations.

To normalize animal numbers to biomass, equivalent animal units were calculated (equal to a 1000 lb or 453 kg animal). Conversion factors are reported in Online Resources Table S1.

The most recent inventory of CAFOs, as of 2018, as well as the percent of which are permitted, were obtained from the US EPA (https://www.epa.gov/sites/production/files/2019-09/documents/cafo_tracksum_endyear_2018.pdf). As noted by the US EPA in reporting these statistics, these numbers include all CAFOs with numbers of animals above the size thresholds set out for large CAFOs. National maps of CAFOs were obtained from Food and Water Watch (2015, 2020). Changes in CAFOs from 2011 to 2017 were also obtained from Walljasper (2018, https://investigatemidwest.org/2018/06/07/large-animal-operations-on-the-rise/).

Manure inventories were obtained from multiple sources. Data from 1982 to 2001 were obtained from Ruddy et al. (2006; the US Geological Survey, https://water.usgs.gov/pubs/sir/2006/5012/excel/Nutrient_Inputs_1982-2001jan06.xls). The US EPA has reported manure N and P by state for the year 2007 (www.epa.gov/nutrient-policy-data/estimated animal-agriculture-nitrogen-and-phosphorus-manure). Sabo et al. (2019) provided manure N estimates for the years 2002, 2007, and 2012 for N by hydrologic unit, and a similar analysis for P was obtained from the US EPA (10.23719/1504278). These latter data were not exclusive to cattle, dairy, broilers and turkeys, but were used to convey trends. These data were herein aggregated by state and then by US region. The most recent animal inventories (2019) were used to calculate the current manure inventory. It is recognized that estimates of animal N and P manure content vary widely, and thus 2 different estimates were applied herein. Estimates of N and P content in manure of each animal type as reported by Ruddy et al. (2006; Online Resources Table S2) are applied to be consistent with older estimates, and more recent manure production factors reported by Bouwman et al. (2017; On line Resources Table S2), are also reported.

Emissions of NH_3_ from fertilizer use and from livestock were obtained from the US EPA National Emissions Inventory (NEI) data (https://www.epa.gov/air-emissions-inventories/2017-national-emissions-inventory-nei-data). The US EPA and the US Agriculture and Forestry Service have reported summaries of greenhouse gas emission trends due to agriculture (www.epa.gov/sites/production/files/2019-04/documents/us-ghg-inventory-2019-main-text.pdf; USDA 2016). Detailed methodology as well as sources of error in analysis are described in the source data reports. Estimates of NH_3_ emissions by animal sector vary widely and represent the composite emissions from animal houses, manure management and land application, and depend on diet, temperature, other environmental conditions and local management practices. To estimate the contribution by animal sector for the most recent animal inventories (2019), emission factors of Bowen and Valiela (2001; Online Resource Table S2) were applied for cattle, dairy, hogs and broilers. It has been suggested (Pelton et al. 2020) that due to the increase in the size of chickens being grown over the past decade, emissions factors for broiler chickens are probably closer to double these earlier estimates. For turkeys, the emission factor reported by the Committee on the Environment and Natural Resources (2000; https://www.esrl.noaa.gov/csl/aqrsd/reports/ammonia.pdf) was applied. Note that the latter source also reports emission factors for other animal sectors, but to be conservative, the former values were applied herein.

Human population was obtained from www.worldpopulationreview.com/states/. Wastewater infrastructure needs by state were obtained from www.infrastructurereportcard.org. Human wastewater N and P were obtained from Sabo et al. (2019) and US EPA (10.23719/1504278), respectively, based on the years 2002, 2007, and 2012.

Comparisons across regions of the US are based on 10 regions of the US as defined by the Office of Management and Budget (OMB; https://www.gao.gov/assets/120/119653.pdf; Online Resource Fig. S1).

## Results

### Farm inventories

**Fig. 2:**
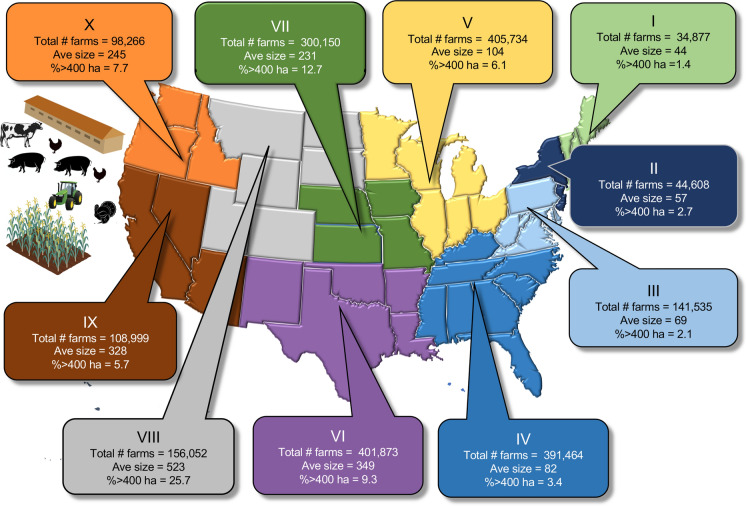
Farm inventory (as total number of farms, average size (ha), and percent with > 400 ha by region of the country. Data are based on 2012 and are summarized from https://cropinsuranceinamerica.org/in-the-states/. The 10 regions of the US are as designated by the Office of Management and Budget (see also Online Resources Fig. S1). Note that Hawaii is included in Region IX and Alaska in Region X. The farm icons are from the UMCES-IAN image library

As of 2012, there were just over 2 million farms in the US. Farms in the northeast and mid-Atlantic (Regions I, II and I II) are the smallest, averaging from 44 to 69 ha per farm with < 2.7% of them of a size exceeding 400 ha (Fig. 2; Online Resource Fig. S2). Farms were somewhat larger in the southeast and upper Midwest (Regions IV, V), averaging 82–104 ha per farm, with 3.4–6.1% exceeding 400 ha. In all of the other regions of the country, farm sizes averaged > 200 ha per farm with largest farms comprising 5.7–25.7% of farms. While there were still over 2 million farms in 2017, the number was down by 12,000 from the previous year, and the average farm size has increased 0.8 ha farm^−1^ year^−1^ since 2012 (www.usfarmdata.com/percentage-of-small-medium-and-large-farms-in-the-us).

### Fertilizer trends with time

**Fig. 3:**
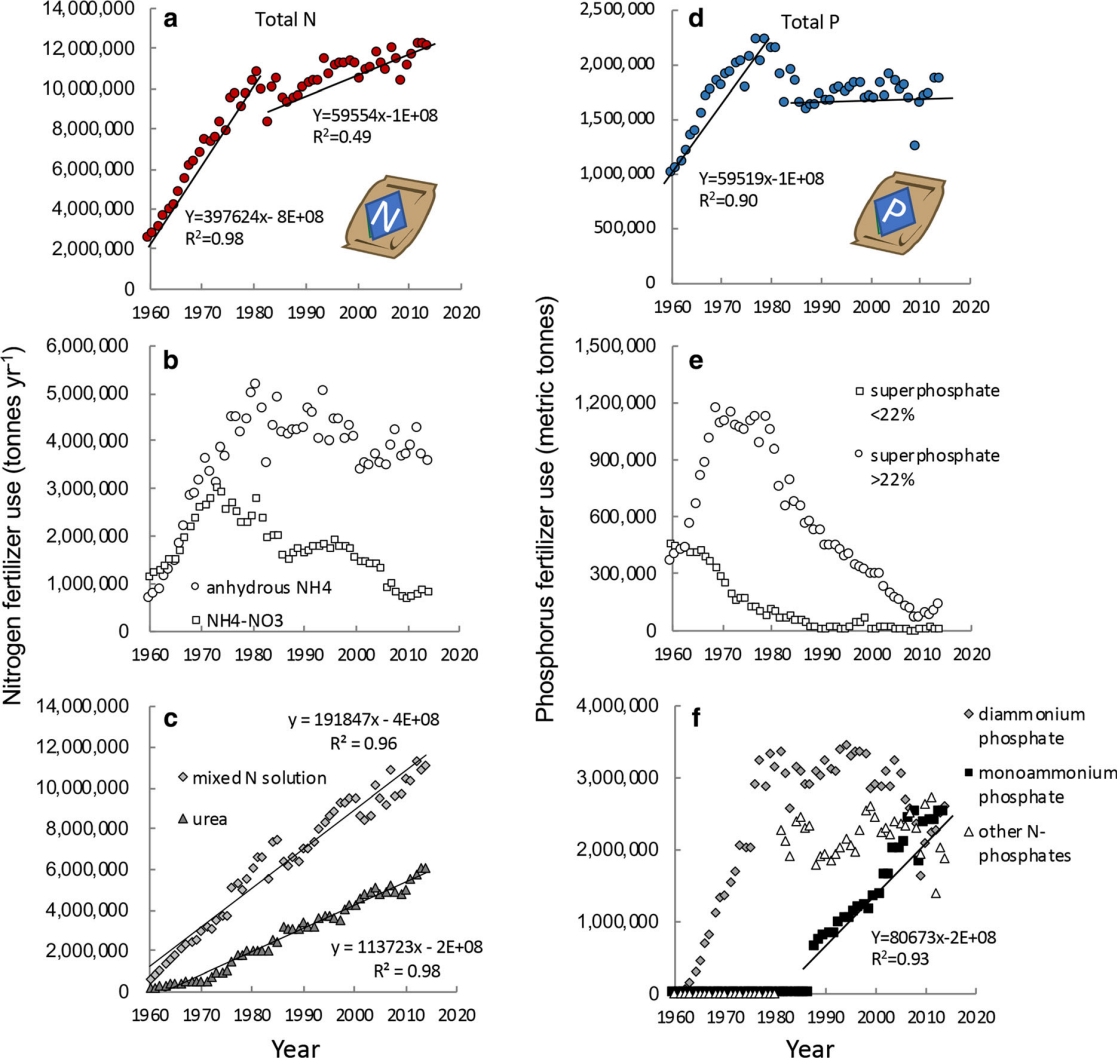
Change in nitrogen and phosphorus fertilizer use in the US over time as **a** total nitrogen, **b** anhydrous NH_4_ and NH_4_–NO_3,_ **c** mixed N solutions (urea-NH_4_–NO_3_ and urea), **d** total phosphorus (as P), **e** superphosphates, and **f** combined N-phosphorus solutions. Data are from https://www.ers.usda.gov/data-products/fertilizer-use-and-price. Trend lines are shown to highlight specific relationships described in text. Icons are from the UMCES-IAN image library

From 1960 to 1980, use of N-based fertilizers in the US increased linearly (r^2^ = 0.98), with nearly 400,000 MT more used year^−1^ (Fig. 3a). From 1980–1990, there was a slight dip in usage, but after 1990 use of N fertilizers increased again, at a slower rate, with only ~ 60,000 MT added year^−1^ (r^2^ = 0.48; Fig. 3a). The current rate of N use is ~ 12 million MT year^−1^ (Figs. 3a).

The formulation of these N fertilizer has changed with time. Use of NH_4_NO_3_ declined sharply after 1970, and that of anhydrous NH_4_ declined after 1980 (Fig. 3b). Use of urea and that of other mixed N solutions (urea-NH_4_–NO_3_) have both shown steady increases since 1960 (r^2^ = 0.98 and 0.96, respectively (Fig. 3c).

For P, as with N, the most rapid rate of increase was from 1960 to 1980, with ~ 60,000 MT of additional P fertilizer used each year (r^2^ = 0.90; Fig. 3d). After a decline from 1980 to 1990, the rate of P use year^−1^ has remained essentially unchanged (slope = 0.0). The current rate of P use is ~ 1.8 million MT year^−1^.

Phosphorous fertilizers also have changed in composition with time. The use of superphosphates, which were common prior to the 1970s, has declined sharply (Fig. 3e). The most recent years have seen a shift to combined N and P forms, of which monoammonium-P use has increased most rapidly; since 1990 its use has increased at the rate of ~ 80,000 MT year^−1^ (r^2^ = 0.93), while use of other forms of P have remained essentially flat or have declined (Fig. 3f).

### Fertilizer trends by crop

**Fig. 4:**
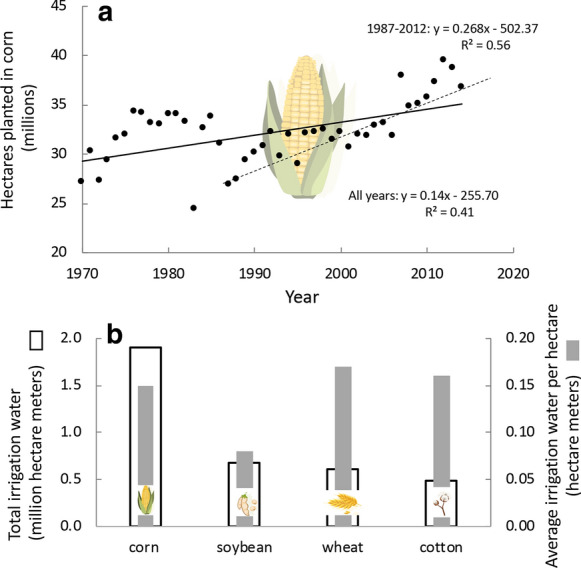
**a** Hectares planted in corn in the US over time. Trend lines are for time period indicated. Data are from https://www.ers.usda.gov/data-products/fertilizer-use-and-price. **b** Irrigation water applied and per ha water use by crop. Data are from Barton and Clark (2014) based on the USDA 2008 Census of Agriculture. Icons are from Vectorstock used under an expanded license

**Fig. 5:**
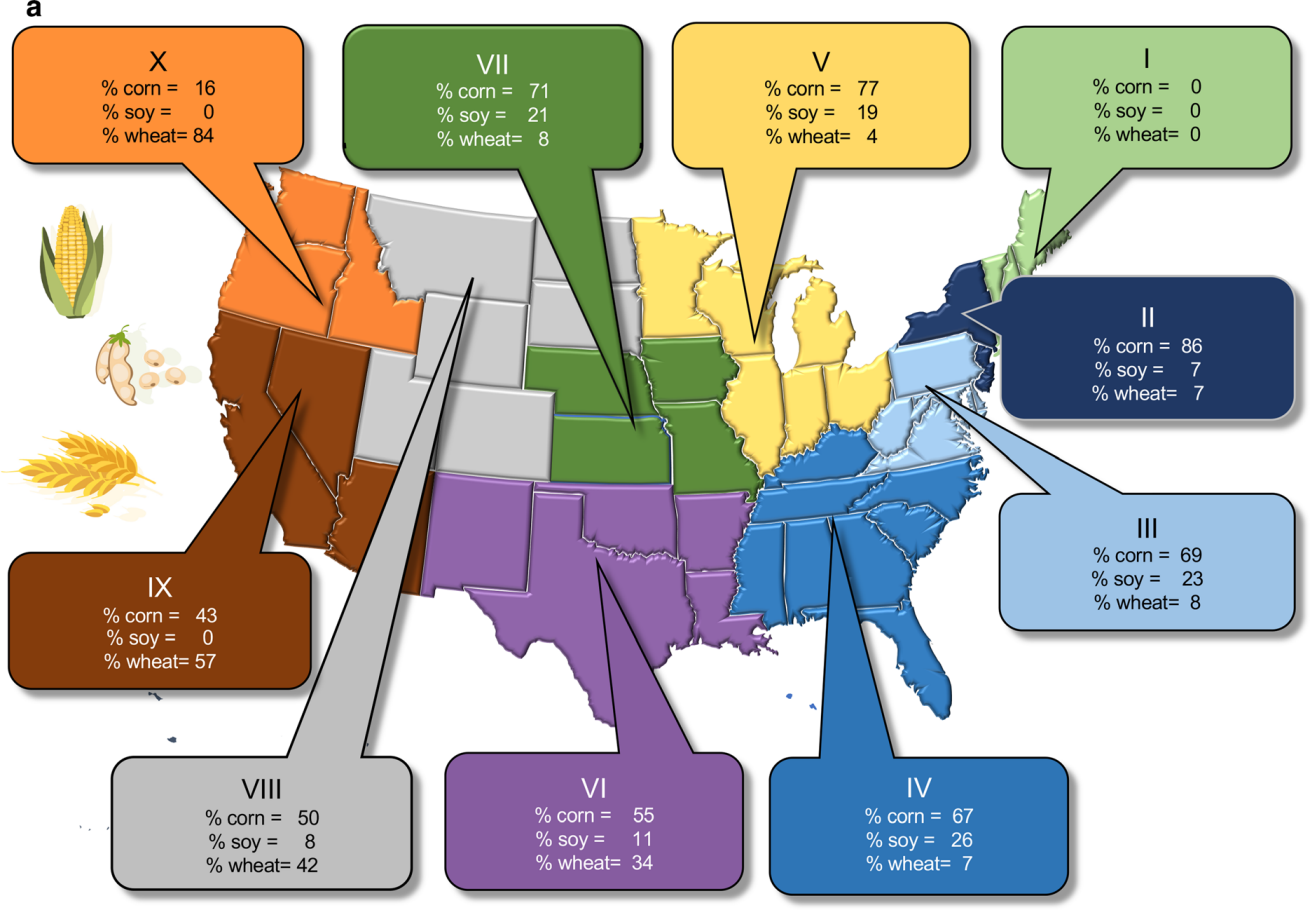

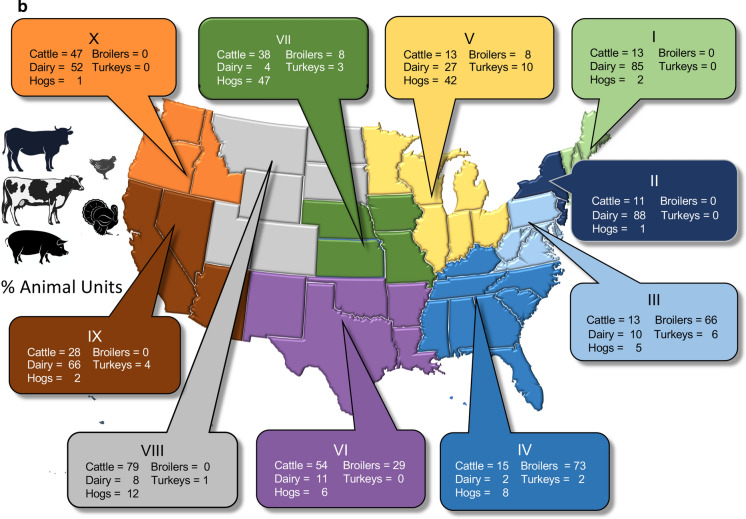
**a** Percent of corn, soybean and wheat grown in the 10 regions of the US designated by the Office of Management and Budget (see also Online Resources Fig. S1). **b** Percent of cattle, dairy, hogs and poultry production for the same US regions, as based on equivalent animal units (see text for explanation). Data are from 2019 from https://www.nass.usda.gov/Statistics_by_State/index.php. Symbols and icons are from Vectorstock used under an expanded license

**Fig. 6:**
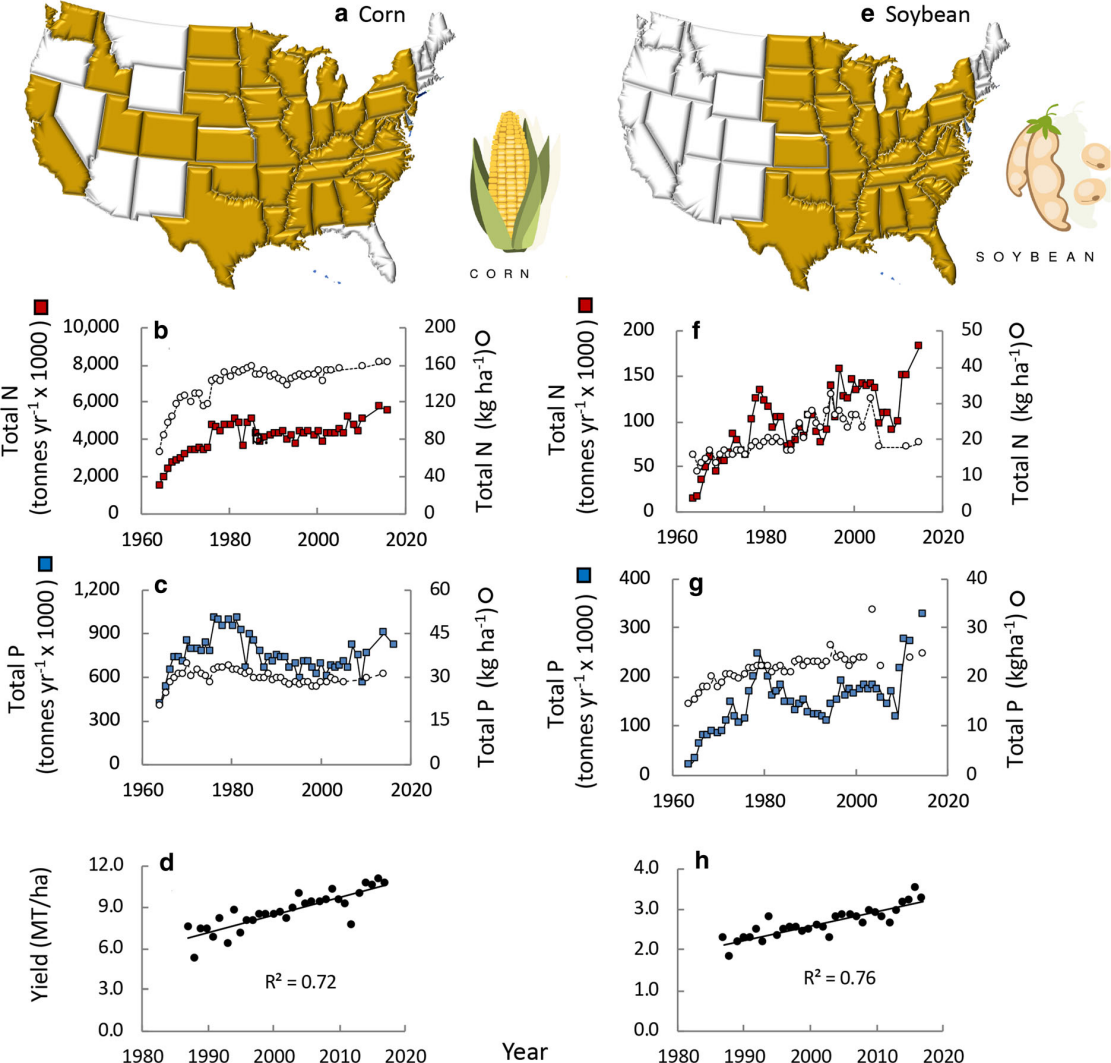
**a** States growing corn, **b** total N fertilizer used on corn over time (squares) and amount per ha (circles); **c** total P fertilizer used on corn (squares) and amount per ha (circles), **d** yield of corn per hectare; **e–h** comparable relationships for soybean. Data are from https://www.ers.usda.gov/data-products/fertilizer-use-and-price. Symbols used are from Vectorstock used under an expanded license

Corn is king, with over 37 million ha planted in this crop as of 2019 (Fig. 4a), yielding 300 million MT (www.nass.usda.gov/Statistics_by_State/index.php, Gunderson et al. 2018). Acreage of corn has increased since the 1970s, and while there was a decline in the early 1980s, there has since been a steady upward trend. Of the three major crops (corn, soybean, and wheat), corn makes up 43–86% of the harvest throughout the country except for the northeast and northwest regions (Regions I and X; Fig. 5a). There are very few states where corn is not grown on an industrial scale (Fig. 6a).

**Fig. 7:**
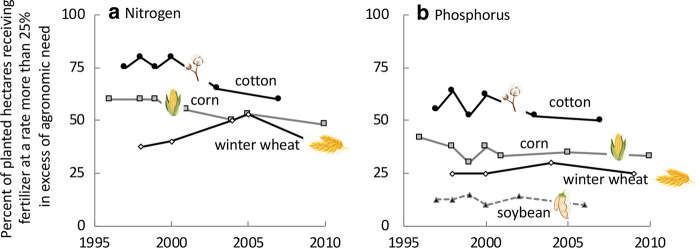
Percent of hectares planted in crop indicated receiving **a** nitrogen or **b** phosphorus fertilizer more than 25% above the recommended agronomic need of the plant. Replotted from https:www.ers.usda.gov/topics/farm-practices-management/crop-livestock-practices/nutrient-management/. Symbols used are from Vectorstock used under an expanded license

Corn is also the most intensively fertilized crop. From the 1990s to present, N fertilization rates for corn have hovered in the range of 140–160 kg ha^−1^ or a total of over 5,500,000 MT year^−1^ (Fig. 6b). As is the case with all crops considered here, fertilizer is often used at a rate that exceeds the agronomic demand by more than 25%; this is to ensure the best yield under ideal conditions. From 1996 to 2010 (most recent data available), for more than 50% of crops planted, the rate of N application was greater than 25% above the plant’s agronomic need (USDA 2019; https://www.ers.usda.gov/topics/farm-practices-management/crop-livestock-practices/nutrient-management/; Fig. 7a). Use of P on corn declined after the 1970s, but has increased about 10% from 2000 to 2014 to 823,000 MT year^−1^ or ~ 30 kg ha^−1^ (Fig. 6c). For 25–50% of crops planted (1996–2010), the rate of P application was greater than 25% above the plant’s agronomic need (USDA 2019; https://www.ers.usda.gov/topics/farm-practices-management/crop-livestock-practices/nutrient-management/; Fig. 7b). The yield of corn has steadily risen from the mid-1980s, with just over 10 MT ha^−1^ now produced (Fig. 6d). Corn also uses the most water for irrigation, although on a ha^−1^ basis, it is comparatively more efficient than other crops considered herein (Fig. 4b).

Soybean, also grown in the Midwest and eastern states (Fig. 6e), makes up 7–26% of the harvest of the three major grains except in the northeast and west coast (Regions I, IX, X), (Fig. 5a). Over 100 million MT are harvested annually (www.nass.usda.gov/Statistics_by_State/index.php). As a legume, it does not need much N fertilization (except in early growth stages), and the amount of N applied to soybeans declined from a peak in the late 1990s, but has risen again in the most recent years, to 184,000 MT (Fig. 6f). Use of P has remained nearly constant in the range of 20–25 kg ha^−1^ over the recent decades, but a spike in P application to 329,000 MT was observed in the most recent years (Fig. 6g). For 10–15% of crops planted (1996–2010), the rate of P application was greater than 25% above the plant’s agronomic need (USDA 2019; https://www.ers.usda.gov/topics/farm-practices-management/crop-livestock-practices/nutrient-management/. Fig. 7b). Yields of soybean, like those of corn have steadily increased over time (Fig. 6h). Soybean are among the most water efficient crops on a ha^−1^ basis (Fig. 4b).

**Fig. 8:**
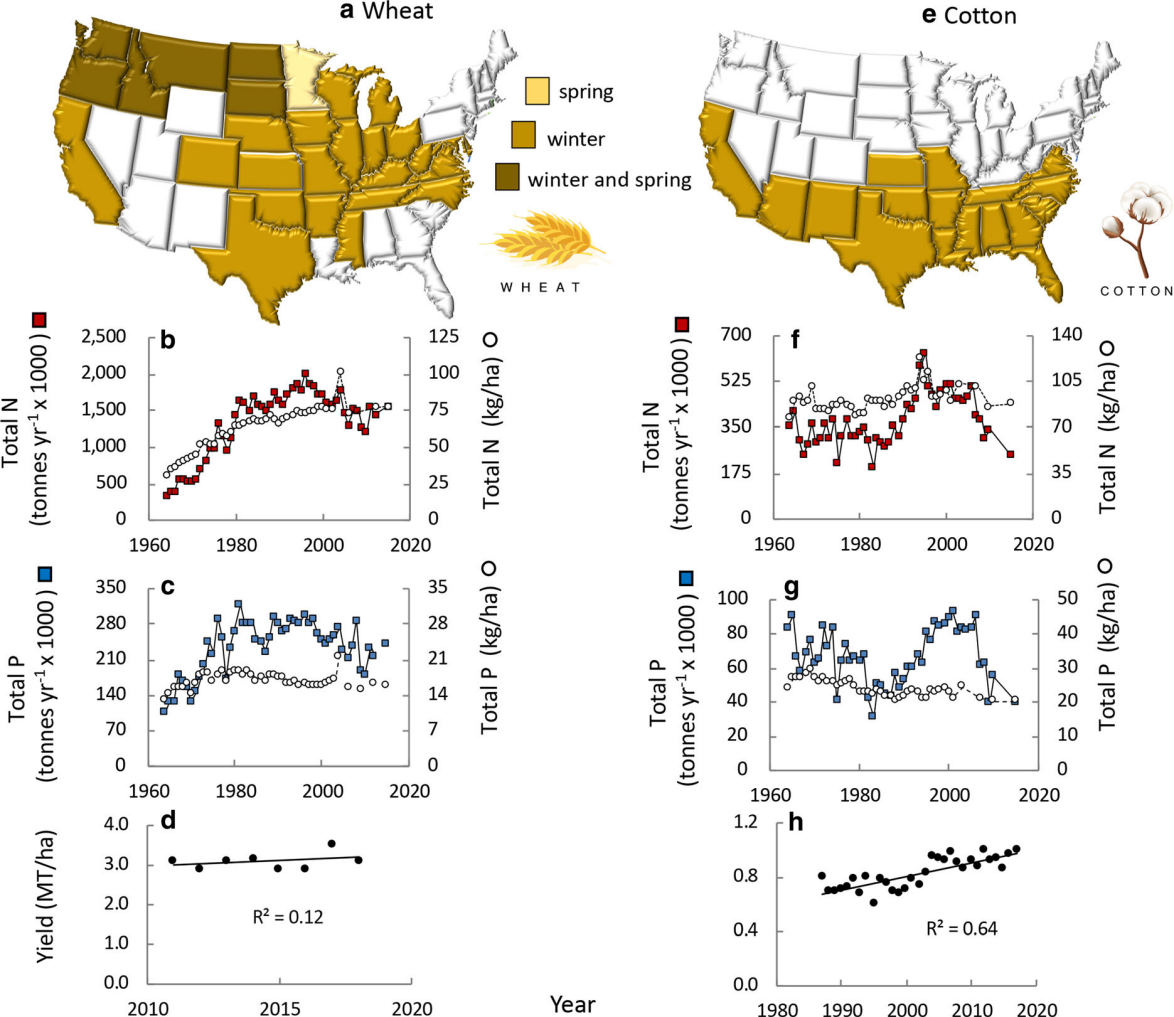
As for Fig. 6 except for **a**–**d** wheat and for **e**–**h** cotton

Wheat is grown throughout the US. In the upper northwest, where both winter and spring crops are planted (Fig. 8a), it makes up 84% of the major crops harvested (Fig. 5a). Over 40 million MT are harvested annually (www.nass.usda.gov/Statistics_by_State/index.php). Use of N on wheat has more than doubled over the decades, from ~ 30 kg ha^−1^ in the 1960s to 78 kg ha^−1^ most recently, with a total N application of 1,437,000 MT (Fig. 8b). For 35–50% of crops planted (1996–2010) the rate of N application was greater than 25% above the plant’s agronomic need (USDA 2019; https://www.ers.usda.gov/topics/farm-practices-management/crop-livestock-practices/nutrient-management/; Fig. 7a). Use of P on wheat reached a peak in the late 1970s, and has declined slightly since then, now at a rate of 242,000 MT (Fig. 8c). For approximately 25% of crops planted (1996–2010), the rate of P application was greater than 25% above the plant’s agronomic need (USDA 2019; https://www.ers.usda.gov/topics/farm-practices-management/crop-livestock-practices/nutrient-management/; Fig. 7b). Data on yields for the past decade reveal little change (Fig. 8d). Wheat requires about twice the amount of irrigation water on a ha^−1^ basis than does soybean (Fig. 4b).

Cotton is grown in the southern states (Fig. 8e). Applications of N to cotton have remained at roughly 100 kg ha^−1^ for the past decades (Fig. 8f), a rate of N application that was more than 25% above the plant’s agronomic need for more than 65% of crops (through 2007; USDA 2019; https://www.ers.usda.gov/topics/farm-practices-management/crop-livestock-practices/nutrient-management/; Fig. 7a). Use of P on cotton has steadily declined from > 60 kg ha^−1^ in the 1960s to 45 kg ha^−1^ most recently, with the most recent application being a total of 39,000 MT (Fig. 8g). Application rates are more than 25% above the plant’s agronomic need for more than 50% of crops planted (through 2007; USDA 2019; https://www.ers.usda.gov/topics/farm-practices-management/crop-livestock-practices/nutrient-management/; Fig. 7b). Yields of cotton have also increased over time (Fig. 8h). Cotton requires comparatively slightly more irrigation water than corn on a ha^−1^ basis, but its overall irrigation demands are far less due to the overall planted acreage (Fig. 4b).

### Fertilizer trends by region and state

**Fig. 9:**
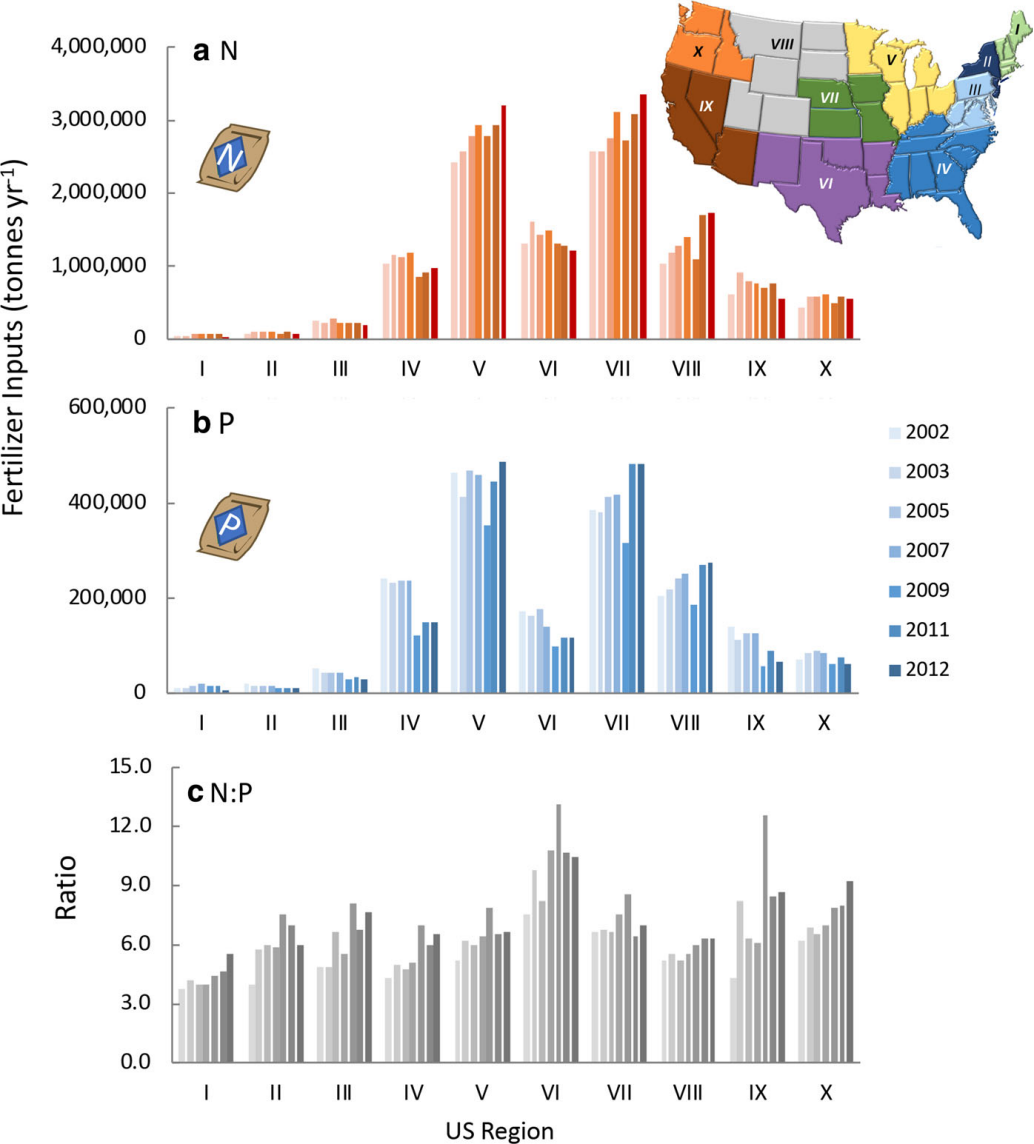
**a** Nitrogen fertilizer purchased by region of the country from 2003 to 2012 and percent change (**b**) As for (**a**) except for P fertilizer. Data from 2003 to 2011 are from the US EPA (https://www.epa.gov/nutrient-policy-data/commercial-fertilizer-purchased). Data for 2002 and 2012 for N were obtained from Sabo et al. (2019), and data for P for the same years are from US EPA (10.23719/1504278). **c** The ratio of N:P (by weight) for the same years. The 10 regions of the US are as designated by the Office of Management and Budget (see also Online Resources Fig. S1). Note that Hawaii is included in Region IX and Alaska in Region X

Regions V and VII are the most heavily fertilized regions, and fertilizer application rates for these regions increased by 32% and 31% for N and by 4.3% and 25% for P from 2002 to 2012 (Fig. 9a,b). Although overall application rates are less in Region VIII, the rate of increase from 2002 to 2012 of both N and P was greater, 64% and 34%, respectively (Fig. 9a,b). Application rates of N and P declined in Regions IV, VI, and IX over this same period. In every region of the US, the N:P of fertilizer application increased from 2002 to 2012 (Fig. 9c).

State-by-state fertilizer use statistics are summarized in the Online Resources material based on 2011 data (Online Resource Fig. S3). Iowa applies N and P more intensively than any other state. As of 2011, its rate of N use was > 1.2 million MT year^−1^, while its rate of P use was ~ 200,000 MT year^−1^. In addition to Iowa, the top states in terms of N usage include Illinois, Nebraska, California, and Minnesota, while the top states for P fertilizer use include, in addition to Iowa, Minnesota, Illinois, Nebraska, and South Dakota.

### Animal operations

**Fig. 10:**
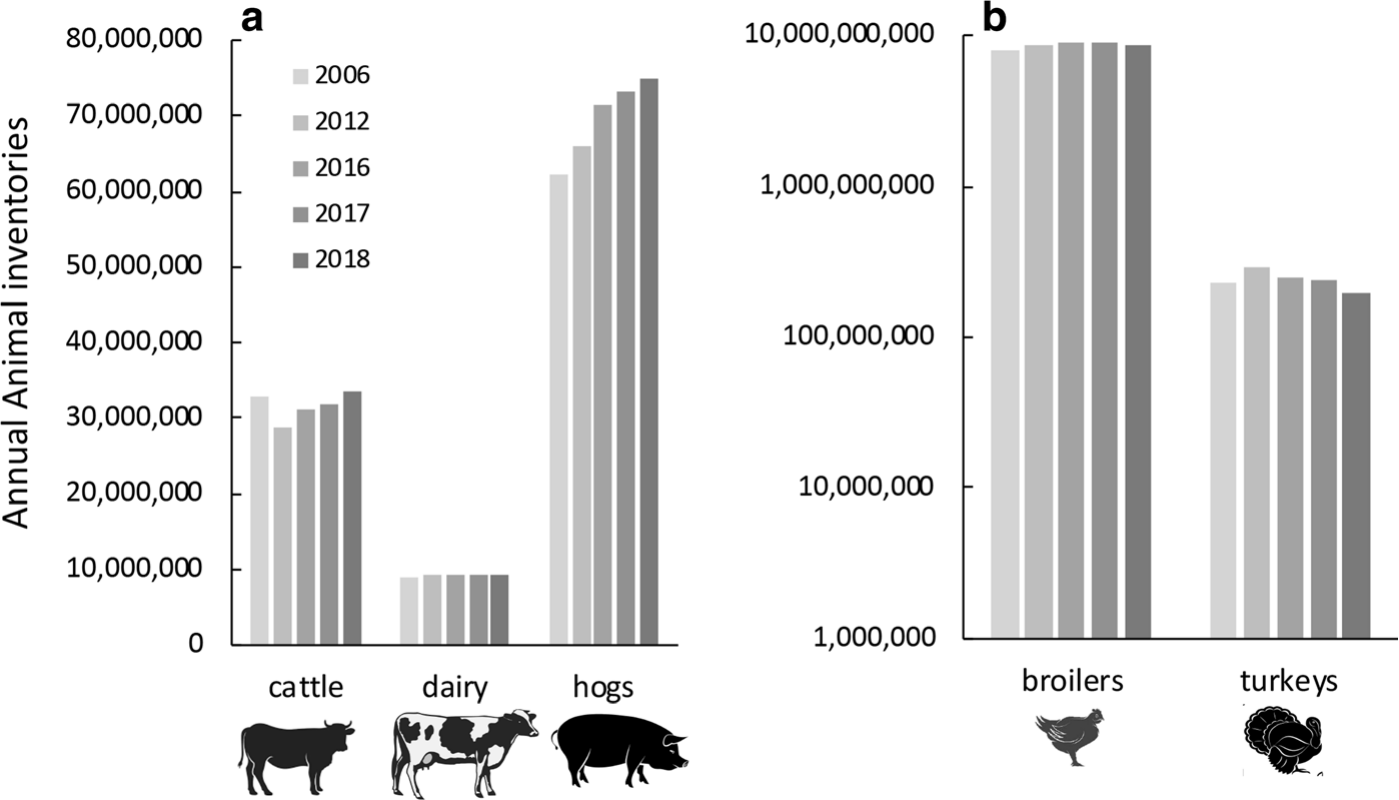
Change in the number of animals by type in medium and large-sized CAFOs in 2019. **a** Numbers of cattle, dairy cows and hogs, and **b** broiler chickens and turkeys. Note the log scale for panel (**b**). Data from 2019 are from USDA (www.nass.usda.gov/Statictics_by_State/index.php). Symbols used are from Vectorstock used under an expanded license

In 2019, the US produced approximately 8.7 billion animals annually in CAFOs, the vast majority being in chickens (Fig. 10a, b). In the 15 years from 1997 to 2012, the number of cattle (on farms with > 500 head) increased 4.3%, dairy cows (on farms with > 500 head) increased 121%, hogs (on farms with > 1,000 head) increased 37%, broiler chickens (on farms producing > 500,000 chickens annually) increased 80% and layers (on farms with > 100,000 hens) increased nearly 25% (Food and Water Watch 2015). This was a net increase of approximately 1 million cattle, 300,000 dairy cows, nearly 14 million hogs and over 250 million broilers, or the equivalent to adding 550 animals every day for 15 years, for hogs adding 3,000 animals every day for 15 years, and for broiler chickens, adding 85,000 chickens every day for 15 years (Food and Water Watch 2015). From 2012 to 2019 cattle increased 13%, dairy cows and broiler chickens ≤ 1%, while hog production increased 13%. During this same time, turkey production decreased 30% (Fig. 10). Thus, the increase in hog production proceeded at about the same rate as pre-2012, adding the equivalent of 3,000 animals or more per day from 2012 to 2019.

Based on animal units, dairy production dominates in the northeast (Regions I,II), broiler production in the southeast (Regions III, IV), hog production in the Regions V, VII, cattle in Regions VI,VII and VIII, while dairy production again dominates in the west (Regions IX, X) (Fig. 5b). State-by-state animal population statistics for 2019 are summarized in the Online Resources material (Online Resources Figs. S4 and S5). Note that these statistics are likely underestimates of the total confined animal populations, as described above (and these statistics do not include populations of animals beyond the groups considered here). Georgia, Alabama, and Arkansas produce over 1 billion broilers annually, Texas has the largest number of cattle, over 4.6 million not including calves, and Kansas, Nebraska, and Texas together account for > 60% of cattle in feedlots (www.aphis.gov/animal_health/nahms/downloads/Demographics2017.pdf). California has the largest number of dairy cows, over 1.7 million (Online Resource Fig. S4), and Iowa has the largest numbers of hogs, with 23 million, outpacing North Carolina, with the next largest populations of these animals, by more than a factor of 2 (Online Resource Fig. S4). The largest region for broiler production is the southeast, with Georgia, Alabama, Arkansas, North Carolina and Mississippi the 5 largest producing states (Online Resource Fig. S5). Turkeys are produced in 13 states, with Arkansas, Minnesota, and North Carolina the largest producers, each with > 20,000,000 animals produced year^−1^ (Online Resource Fig. S5).

**Fig. 11:**
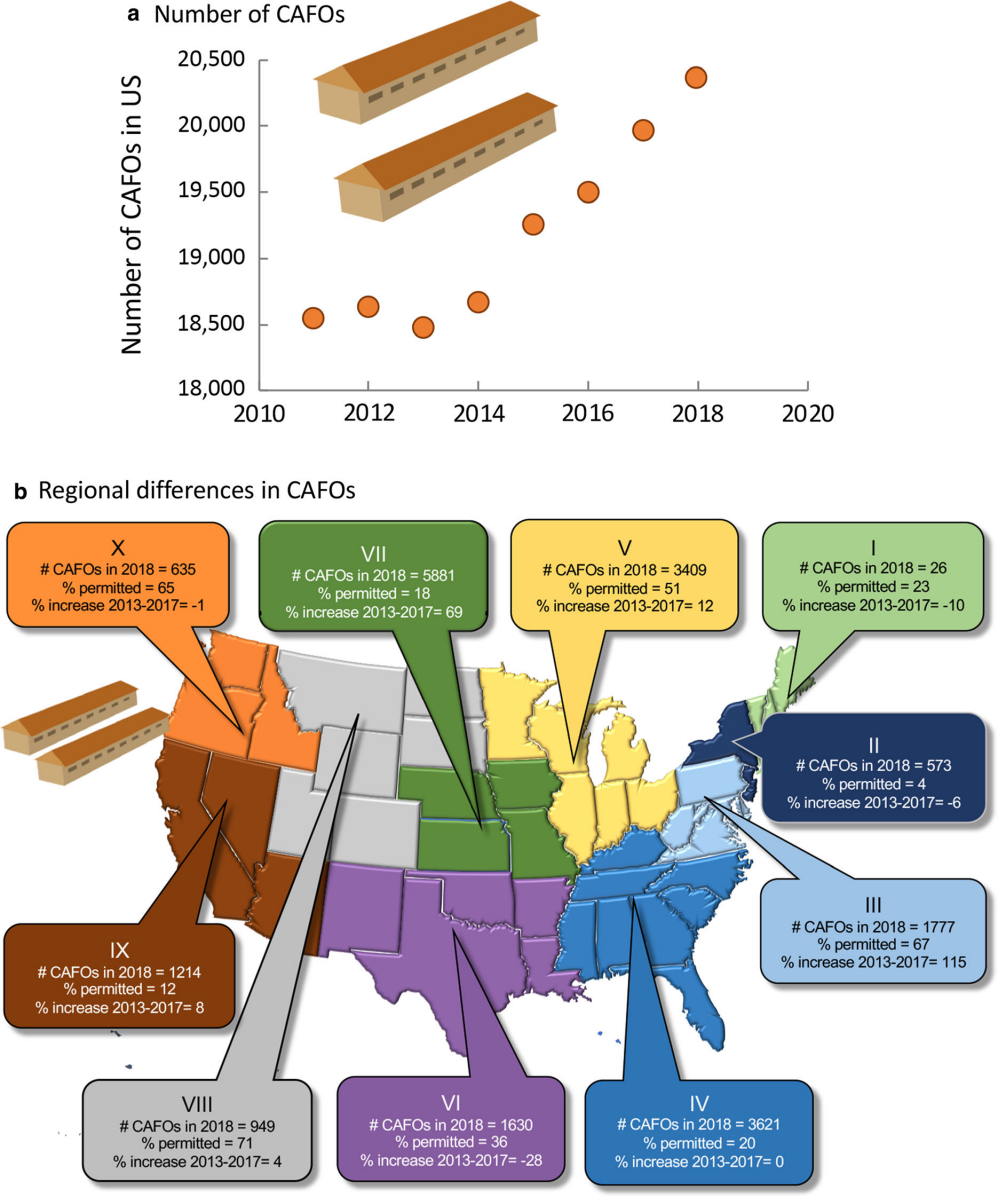
**a** Total US changes in CAFOs from 2011 to 2018, **b** numbers of CAFOs by US region in 2018, their percent change from 2013 to 2017 and percent permitted. Data are from EPA (https://www.epa.gov/sites/production/files/2019-09/documents/cafo_tracksum_endyear_2018.pdf) and USDA as summarized by Walljasper (data 2011–2017, https://investigateMidwest.org/2018/06/07/large-animal-feeding-operations-on-the-rise/). Symbols used are from Vectorstock used under an expanded license. The 10 regions of the US are as designated by the Office of Management and Budget (see also Online Resources Fig. 1). Note that Hawaii is included in Region IX and Alaska in Region X

As of 2018, the US had over 20,000 CAFOs, a number that has increased ~ 8% in the past decade, but a number that likely underestimates the true value (Fig. 11a; Online Resources Fig. S6a). The highest concentration of CAFOs is in Region VII with over 5,800, followed by Regions IV with 3621, and Region V with 3409 (Fig. 11b). The largest expansion in such operations was in Region VII, where 69% more CAFOs, and in Region III, 115% more CAFOs, now operate compared to a decade ago (Fig. 11b). States with over 1000 CAFOs in 2018 include Texas, California, Nebraska, North Carolina, Minnesota, and Iowa, which has the highest number overall, with > 3500 (Online Resource Fig. S6a). States with the largest increases in CAFOs from 2011 to 2018 were Maryland and Delaware, in chickens, and Iowa, in hogs (Online Resource Fig. S6b).

In terms of permitting, the National Pollutant Discharge Elimination System (NPDES, the regulation system authorized by the Clean Water Act) requires that all CAFOs that discharge to a waterbody have NPDES permit coverage (40CFR 122.23(d)(1)). As a consequence, the portion of CAFOs that need NPDES coverage can vary from state to state depending on size, discharge and waste management systems. On average across all states, only 32% of CAFOs are permitted under the NPDES regulations. Regions I, II, IV, VII, and IX had fewer than 20% of operations permitted, while regions III, V, VIII, and X had over 50% of operations permitted (www.epa.gov/sites/production.files/2019-09/documents/cafo_tracksum_endyear_2018.pdf). Iowa, with over 3,700 CAFOs, has permits for just 3%, and North Carolina, with over 1200 CAFOs, has permits for 1%; these are the top 2 states for hog production (Online Resources Fig. 6c). Of the 8 states with the largest CAFOs, 24% have permits. States with higher production of chickens, such as Maryland and Alabama, have much higher permitting percentages.

Cattle operations are concentrated in the Midwest and the largest expansion in cattle CAFOs from 2011 to 2017 were in Missouri and Colorado (Online Resource Fig. S7). Increases in dairy were concentrated in the southwest and upper Midwest, with Texas, Missouri, Colorado, Kansas and South Dakota increasing production by close to, or more than, 20% (Online Resource Fig. S8a–c). Hog production decreased in the southwest but became more concentrated in the upper Midwest from 2011 to 2017 (Online Resource Fig. S8d–f). Virtually every county in Iowa is now in intensive hog production (Online Resource Fig. S8f). Broilers remain concentrated in the southeast, but Ohio increased production by > 50% (Online Resource Fig. S8g-i).

### Manure quantities

**Fig. 12:**
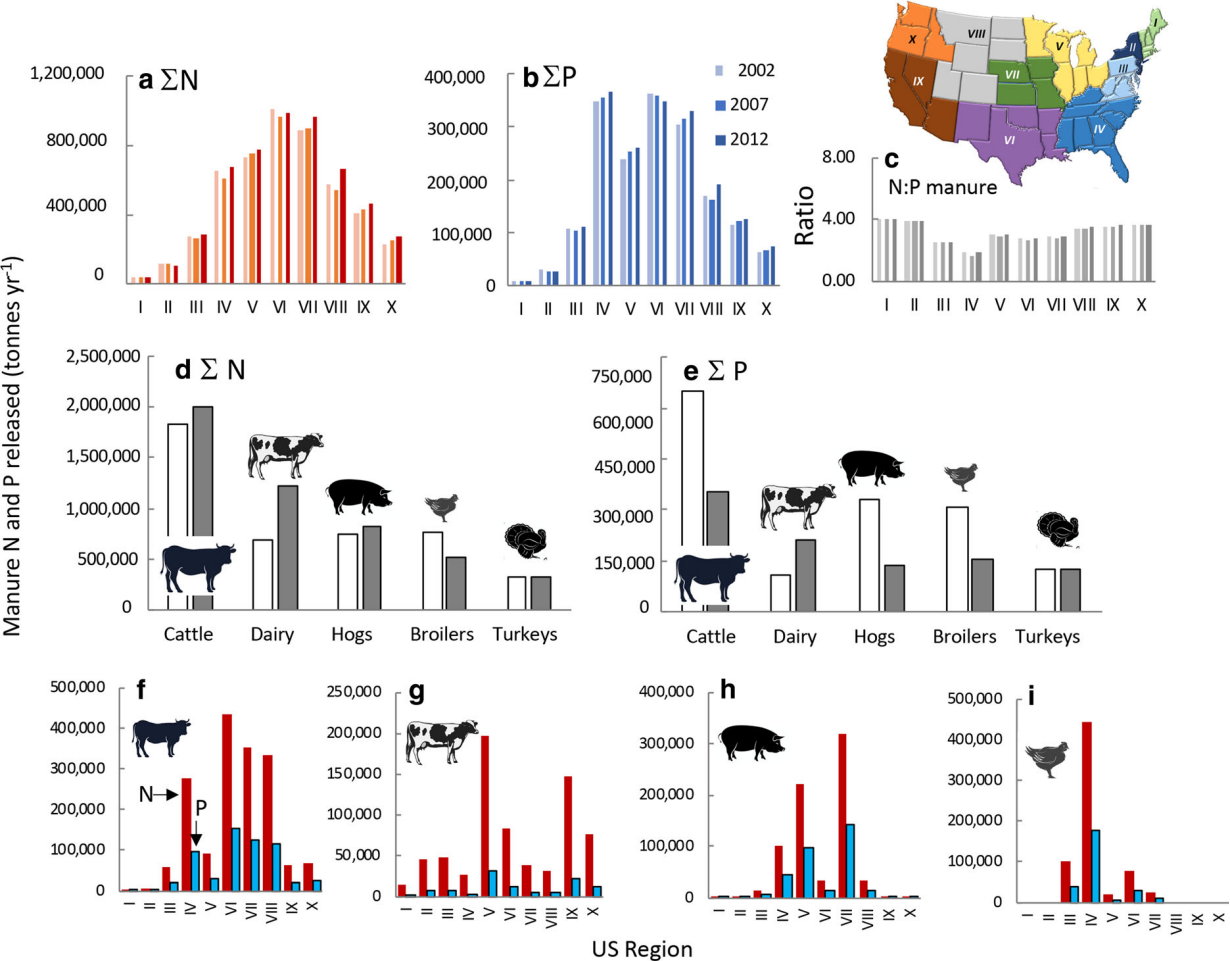
Daily amount of excretion of manure as **a** N, **b** P, and **c** N:P ratio by weight by US region. Data are for 2002, 2007, and 2012 and were derived from Sabo et al. (2019) for N and US EPA (10.23719/1504278) for P. The upper inset map shows the US regions. Panels **d**, **e** are calculated data for N and P released as manure by animal type for 2019 (data from USDA www.nass.usda.gov/Statictics_by_State/index.php). Open bars represent values calculated using conversion factors reported by Ruddy et al. (2006); closed bars represent values calculated using conversion factors reported by Bouwman et al. (2017). Panels **f**–**i** show the same 2019 data by US region (applying Ruddy et al. 2006 conversions). The 10 regions of the US are as designated by the Office of Management and Budget (see also Online Resources Fig. S1). Note that Hawaii is included in Region IX and Alaska in Region X. Symbols used are from Vectorstock used under an expanded license

In most regions of the US, total N and P from manure increased from 2002 to 2012 (Fig. 12a, b). In Regions IV-XIII, > 400,000 MT year^−1^ manure N are released, while in Regions IV–VII, > 200,000 MT year^−1^ manure P are released. The N:P ratio (by weight) of manure is lowest in Regions III and IV (Fig. 12c) and for each region has not changed substantially over this time period.

Based on the animal inventory of 2019, over 4 million MT of manure as N was produced from all animals in confinement considered herein. Applying the conversion factors of Ruddy et al. (2006), ~ 44% was from cattle, ~ 17 18% from dairy cows, hogs, and broilers, and 3.9% from turkeys (Fig. 12d). Applying the conversion factors of Bouwman et al. (2017), the contribution from dairy is nearly twofold higher, that from cattle and hogs slightly higher, while that from broilers ~ 30% lower.

For the same time period, over 1.4 million MT year^−1^ of manure as P was produced. Applying the conversion factors of Ruddy et al. (2006), cattle produced 45%, hogs and broilers each 20–23%, dairy cows nearly 8%, while turkeys just 4.3% of this P (Fig. 12e). The Bouwman et al. (2017) conversion factors yield values ~ 40% lower for cattle, hogs and broilers, but higher values for diary.

Regions IV, VI, VII, and VII produced the most N from cattle, Regions V and IX from dairy cows, Regions IV, V, and VII from hogs, and Regions III and IV from broilers (Regions 12f–i). Regions III, IV and V were the largest turkey production regions (not shown).

### Ammonia emissions

**Fig. 13:**
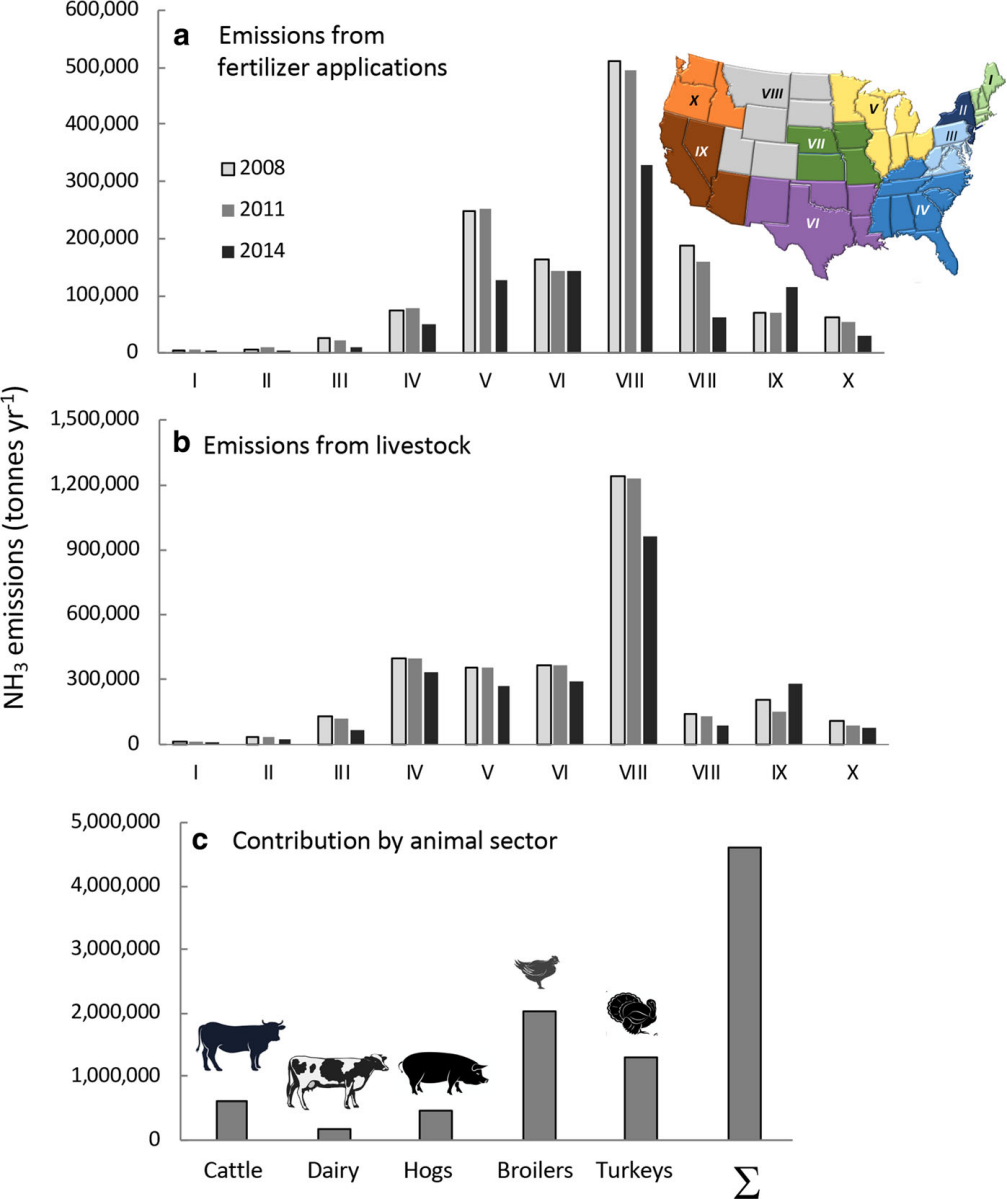
**a** NH_3_ emissions from fertilizer applications and **b** from livestock (as total MT) for different regions of the country and recent changes, and **c** emission for 2014 by animal type. Data were derived from the EPA National Emissions Inventory (NEI) (https://www.epa.gov/air-emissions-inventories/2017-national-emissions-inventory-nei-data). The 10 regions of the US are as designated by the Office of Management and Budget (see also Online Resources Fig. S1). Note that Hawaii is included in Region IX and Alaska in Region X. Symbols used are from Vectorstock used under an expanded license

There are two major sources of NH_3_ emissions from agricultural operations. It is emitted from fertilizer applications, especially when those applications are NH_4_- or urea-based, and from management of manures. Emissions summaries are available by state in the Online Resources (Online Resource Fig. S9; https://www.epa.gov/air-emissions-inventories/2017-national-emissions-inventory-nei-data). Emissions have not only fluctuated with time, generally showing a decline from 2008 to 2014, but the methodology for reporting has changed slightly over time and thus there is high variability in these data from year to year by region (Fig. 13a). Emissions of NH_3_ from fertilizer applications ranged from very low in the northeast to a high of over 300,000 MT year^−1^ in Region VIII in 2014 (Fig. 13a). Region VIII also produces the highest NH_3_ emissions from livestock waste, with values over threefold higher than those from fertilizer applications (Fig. 13b). Based on data from 2014, states with the largest NH_3_ emissions from fertilizer, > 50,000 MT year^−1^, included California, Texas, Kansas and Illinois (Online Resource Fig. S9a), and those with the largest emissions from livestock waste, > 100,000 MT year^−1^, include California, Texas, Iowa, and North Carolina (Online Resource Fig. S9b).

Based on the animal inventory of 2019, a total of > 4,500,000 MT year^−1^ of NH_3_ were emitted (Fig. 13c). Of this, broilers and turkeys made the largest contribution. This value was derived using a conservative emission factor for broilers, and would be significantly greater if a higher emission factor were applied.

### Greenhouse gas emissions

**Fig. 14:**
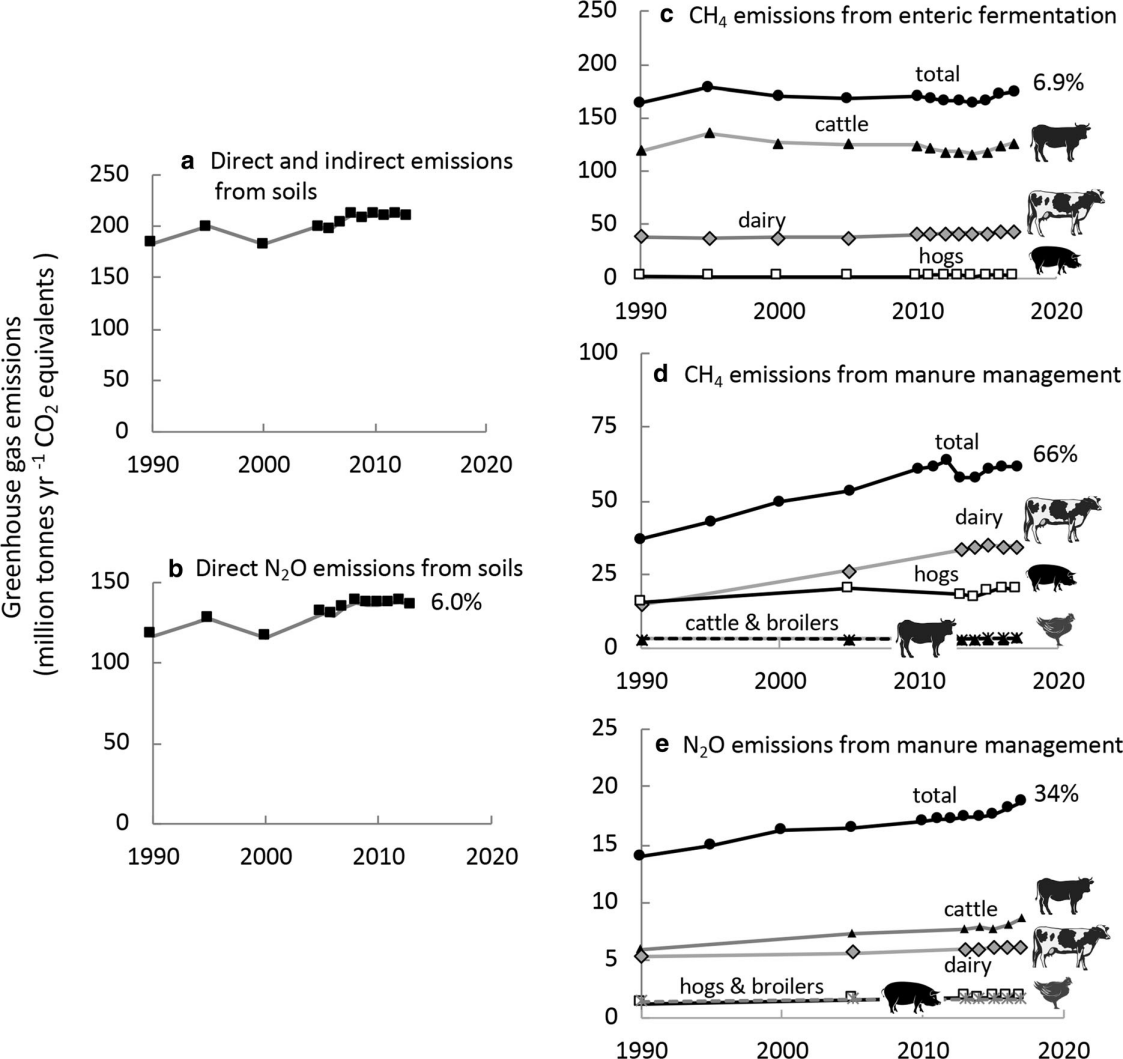
Greenhouse gas emissions as CO_2_ equivalents from **a** direct and indirect sources related to soils: **b** direct N_2_O emissions from soils: **c** from CH_4_ enteric emissions by animal sector, **d** from CH_4_ from manure management by animal sector; and **e** from N_2_O emissions from manure management by animal sector. Data are from EPA (www.epa.gov/sites/production/files/2019-04/documents/us-ghg-inventory-2019-main-text.pdf). Symbols used are from Vectorstock used under an expanded license

In 2017, the agriculture sector emitted 542 million MT CO_2_ Eq (using equivalencies reported by the IPCC Fourth Assessment Report 2007), representing 8.4% of US greenhouse gas total emissions. Direct and indirect emissions, largely as N_2_O from soils, contribute substantially to this agriculture component of greenhouse emissions (Fig. 14a,b). Most of this comes from cropland compared to grassland. Although there are interannual variations, the change from 1990 to 2017 in this source was only 6% (Fig. 14b).

Enteric fermentation accounts for the largest fraction of CH_4_ emissions from the agriculture sector (Fig. 14c). Of the total production of ruminant animals, cattle were the largest contributors from enteric fermentation (Fig. 14c). From 1990 to 2017, there was an increase in total enteric fermentation emissions of 6.9%, and year-to-year fluctuations in emissions per head per type of animal are attributed to changes in animal diets among other factors. In sharp contrast to the comparatively small percentage change in greenhouse gas emissions over the past decade due to enteric fermentation, there has been a sharp rise in greenhouse gas emissions due to manure management. Emissions of CH_4_ from manure management increased 66% from 1990 to 2017 (that from dairy increased 134%, cattle 9.6%, hogs 29% and poultry 3%), while those of N_2_O increased 34% over the same time period (dairy 15%, cattle 46%, hogs 58%, and poultry 14%; Fig. 14d,e).

Texas has the highest greenhouse emissions overall (Online Resource Fig. S10a), while California, Idaho, Iowa and North Carolina have the largest CH_4_ emissions (Online Resource Fig. S10b), with emissions of the first 2 states largely due to dairy and emissions of the latter two states mostly due to hogs. Kansas, Nebraska and Texas have the largest N_2_O emissions due to cattle (Online Resource Fig. S10c).

### Human population and wastewater

As of mid-2019, the US human population was 328,557,738 persons (https://worldpopulationreview.com/states/). California is the most populous state, Wyoming the least (Online Resource Fig. S11a). Since 2010, states that have experienced a > 10% increase in population include Texas, Florida, Washington, Arizona, Colorado, Utah, Nevada and Idaho. Only Illinois, Connecticut and West Virginia have undergone population declines over this period. Due to the size of the state and its large population, wastewater from California’s urban areas contribute more than any other state.

**Fig. 15:**
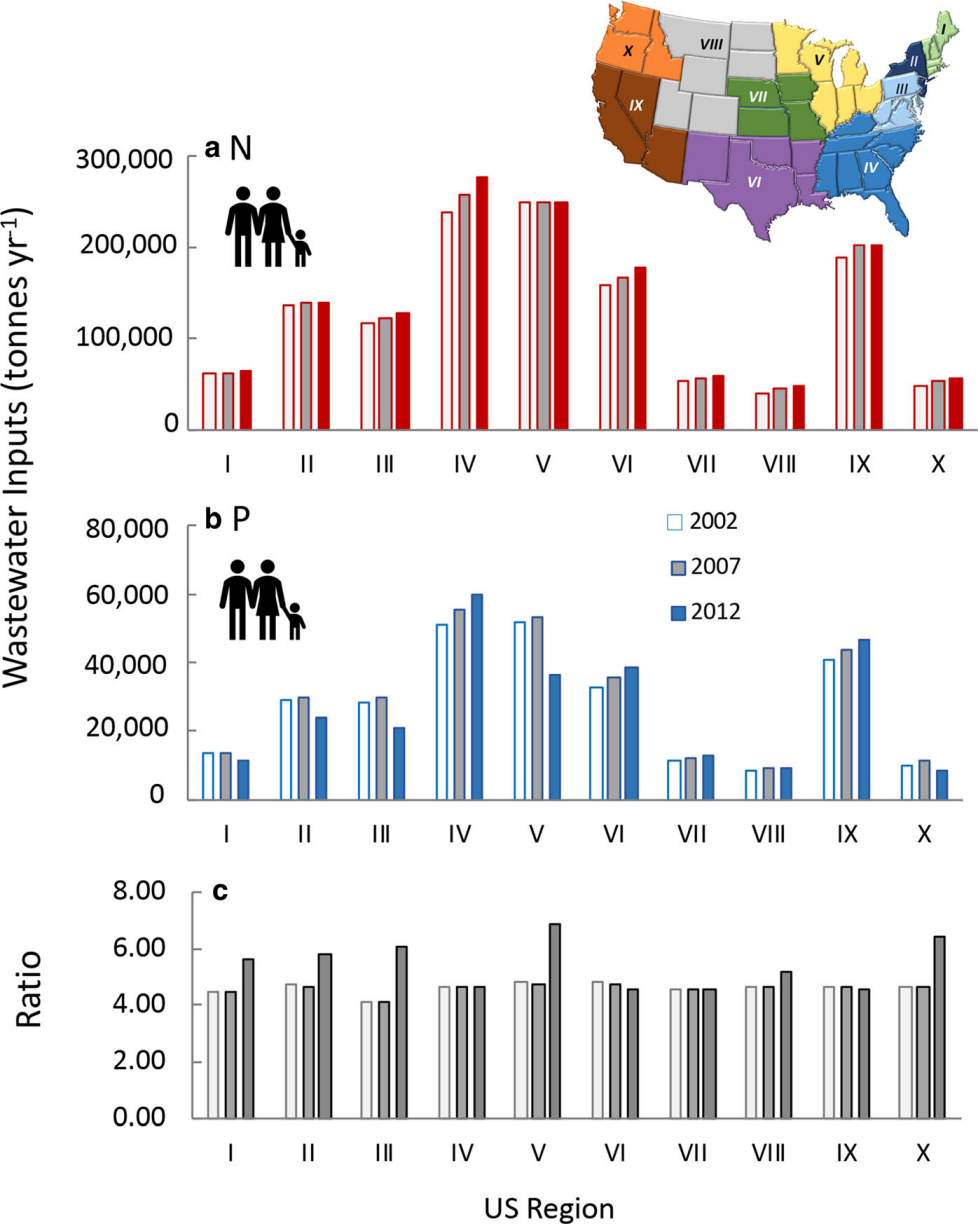
Human wastewater inputs of **a** nitrogen, **b** phosphorus, and **c** N:P ratio by weight for different regions of the country. Data are for 2002, 2007 and 2012 and were derived from Sabo et al. (2019) for N and from US EPA (https://doi.org/10.23719/1504278) for P. The upper inset map shows the US regions

Based on the human wastewater estimates of Sabo et al. (2019) for N and the US EPA for P, aggregated by region, wastes for both elements are highest from Regions IV, V, and IX (Fig. 15a,b; Online Resource Fig. S11b, c). Wastewater N has increased from 2002 to 2012 in virtually all regions, but wastewater P in some regions has declined (Fig. 15b). Accordingly, wastewater N:P proportions increased from 2002 to 2012 in all but Regions IV, VI, VII, and IX (Fig. 15c).

Statistics are also available on the investment needed in wastewater infrastructure by state anticipated over the next 20 years (infrastructurereportcard.org). These data give some clues as to the level of wastewater treatment. States have widely varying infrastructure needs for wastewater treatment in the next 20 years, but overall, those states with the most rapid growing population have proportionately lower estimated infrastructure costs (Online Resource Fig. S11d). California, Texas, Florida, New York Ohio and New Jersey all have needs exceeding $10 million over the next 20 years, but on a per-person basis, the largest costs, > $1500 per person over 20 years, are estimated for New York, New Jersey, Missouri, Maryland, West Virginia, Hawaii, and Rhode Island (Online Resource Fig. S11d).

### Summary comparisons of N and P sources by region

**Fig. 16:**
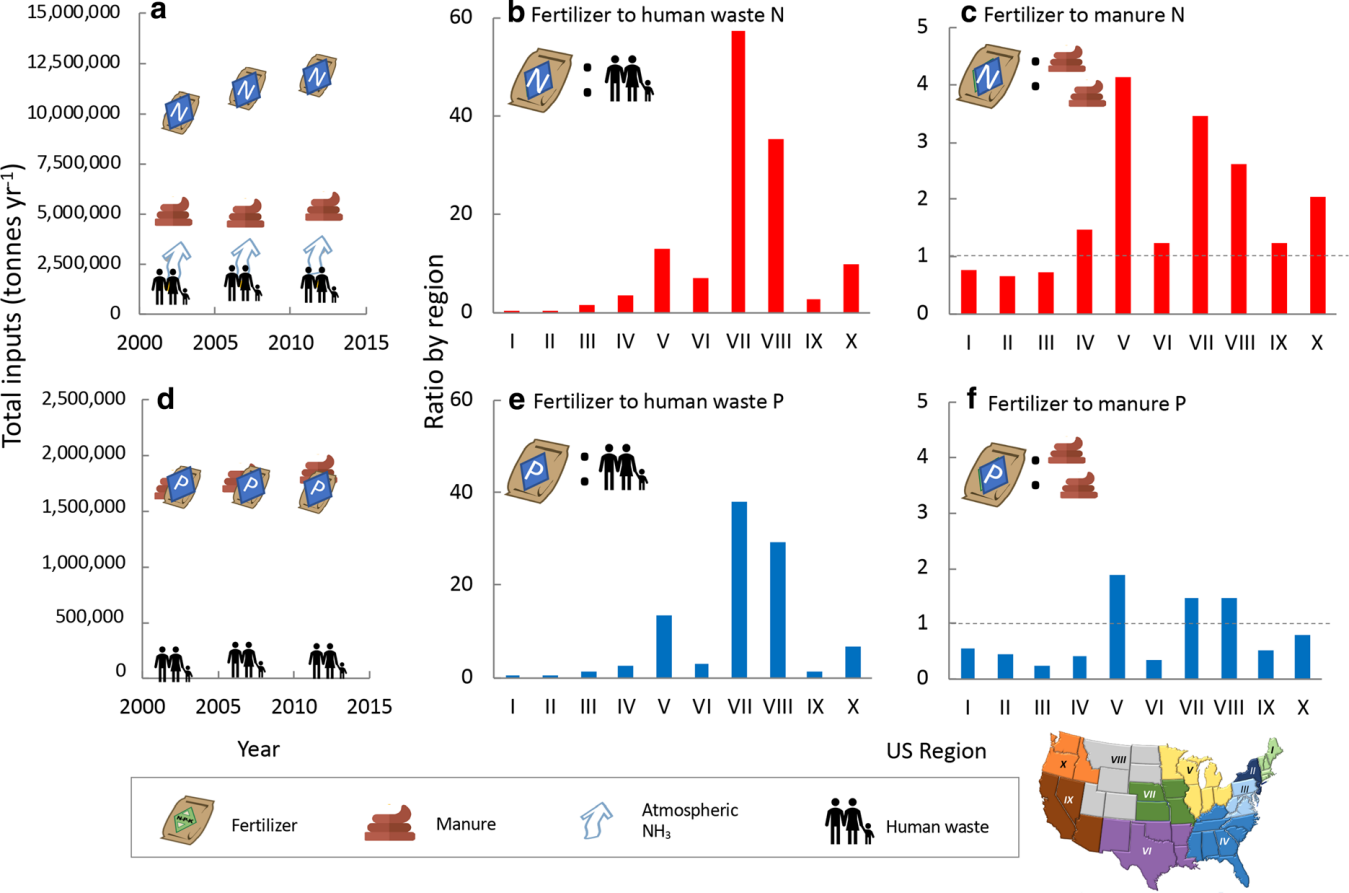
Comparisons of N and P inputs. **a** Recent changes in N fertilizer, manure N, atmospheric NH_3_ and human wastewater for the years 2002, 2007 and 2012 for the entire US. Data were derived from Sabo et al. (2019). Panels **b**, **c** compare fertilizer N to human wastewater N input, and fertilizer N to manure N input, respectively, for different regions of the country. Panel **d** as for panel **a** except for P; data were derived from US EPA (10.23719/1504278). Panels **e**, **f** are the same as panels **b**, **c** except for P. For panels **c**, **f** a dashed line is shown at a ratio = 1 for reference. The inset map shows the US regions

For the country as a whole, fertilizer N inputs have been increasing, and total N inputs from this source are > twofold those of manure N, > threefold those of atmospheric NH_3,_ and nearly tenfold higher than those from human wastewater (Fig. 16a). Regionally for 2012, the proportion of fertilizer N inputs relative to human wastewater are very low in the densely populated mid-Atlantic and northeast, Regions I–III, but reach values in excess of 35 in Regions VII and VIII (Fig. 16b). Also, only in Regions I–III are fertilizer inputs less than those of manure N. In all other regions of the country, fertilizer N inputs exceed those of manure by factors ranging from < 2 (Regions IV, VI, and IX) to as high as 4 in Region V (Fig. 16c).

For P, fertilizer and manure P inputs have been roughly on par since the early 2000s, but manure inputs are increasing, while those of P fertilizer have been declining on a relative basis (Fig. 16d). Both of these sources were far in excess of those from human wastewater in 2012. Regionally, fertilizer P inputs exceed those of human wastewater by factors > 3 in Regions V, VII, VIII, and X; for all other regions this proportion is < 3 (Fig. 16e). Also, only in Regions V, VII, and VIII did P fertilizer inputs exceed those of manure; for all others, manure inputs of P exceed those of fertilizer (Fig. 16f).

## Discussion

### Key trends

**Fig. 17:**
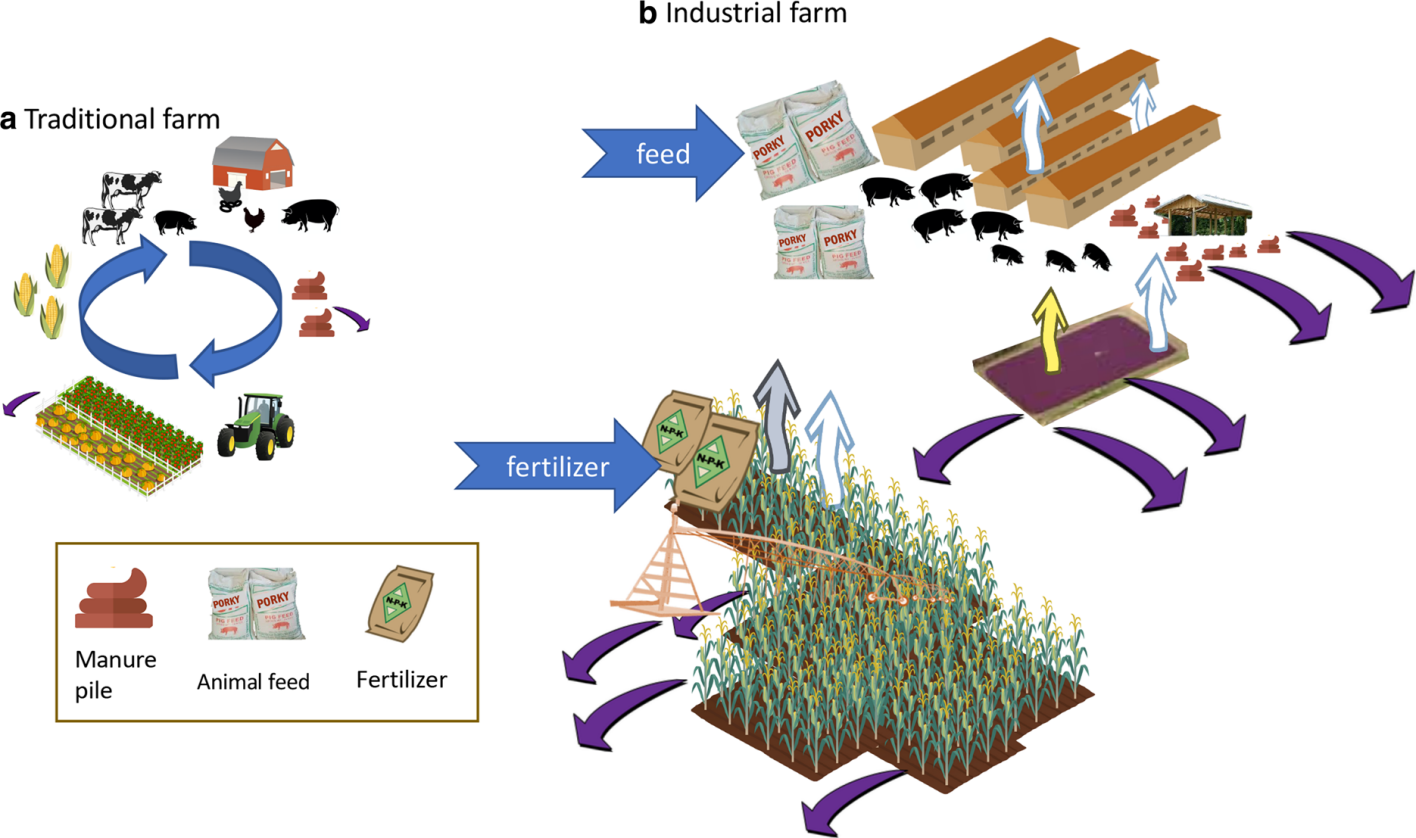
**a** Classically envisioned nutrient cycle of a traditional farm. Wastes from various animals are used to grow seasonally appropriate crops, and some of this food is used in feeding the animals. **b** On industrial farms, animal populations (typically single species) produce copious manure that is held in waste lagoons and spread on fields of a mono-crop, supplemented with fertilizer. Feed for the animals is tightly controlled and imported on the farm. Wastes from fertilizer runoff and manure N are not recycled but rather lost to the environment. Symbols used are from the UMCES-IAN image library or from Vectorstock used under an expanded license

Farmers have long been considered inherently good stewards of the land. The historical balance that small farmers sustained between animal waste production and crops that fed both animals and people is still the notion that many have with respect to farming (Fig. 17a). This ingrained belief has resulted in agricultural operations having the privilege of exemptions of many provisions of environmental laws (Schneider 2010 cited in Tomas 2019). This notion of good stewardship contrasts with current reality and thus, “…rather than reach a middle ground that balanced agriculture and environmental conservation, policy-makers largely yielded to agricultural exceptionalism—nearly every major federal environmental statute passed since 1970 has included carve-outs for farms…” (Ruhl 2000). Now, as the scale of row-crop farms and CAFOs have increased, such good stewardship and environmental nutrient balance within farms can no longer be assumed. Hanson and Hendrickson (2009), citing Stauber et al. (1995) summarized the guiding economic principles of industrialized farming, among which include: “(1) nature is a resource to be exploited and variation is to be suppressed, (2) natural resources are not valued except when a necessary expense in production is incurred, (3) progress is equivalent to the evolution of larger farms and depopulation of farm communities”. Farms are now importing fertilizer for crops and feed for animals and the waste production far outpaces that which can be safely recycled back on the land (Fig. 17b). As noted by Pollan (2006), the classically integrated closed ecological loop on traditional farms has been replaced by a disconnected system with a need for increasing chemical fertilizers to support crops and feed for animals, and a resulting manure waste problem from the feedlot.

The effort here is intended to “step back” and to bring attention to recent trends in nutrient sources and that of CAFO proliferation. This paper is hardly the only voice sounding the alarm on the overwhelming nutrient pollution especially from the expansion of CAFOs (e.g., Mallin and Cahoon 2003; Burkholder et al. 2007; Potter et al. 2010; Sakadevan and Nguyan 2016; Rumpler 2016; Martin et al. 2018; Miller and Muren 2019; Pelton et al. 2020 among others). It has long been recognized that only a small fraction of agriculturally used or produced N and P (as fertilizer or manure) actually reaches human consumers in the intended food products (e.g., Galloway et al. 2002; Houlton et al. 2013), and roughly half is ultimately lost to the environment in direct runoff and indirect pathways such as atmospheric volatilization and eventual deposition (Galloway et al. 2014). Rather than reporting detailed inventories, the focus here is on total inputs as fertilizer, manure, NH_3_ and greenhouse gas production relative to human wastewater. Collectively, this effort—as well as the more comprehensive inventories on which this analysis was based—all underscore that inputs are increasing, nutrient pollution from CAFOs is large and increasing, and highly concentrated in certain regions of the country. Clearly, wastes from the more than annually-produced 8.7 billion animals, mostly confined to nearly 20,000 CAFOs, and 328 million people, combined with roughly 12 million MT of N and 1.8 million MT of P of commercial fertilizer, > 4,000,000 MT of manure as N and > 1,400,000 MT manure as P, along with an estimated > 4,500,000 MT of atmospheric NH_3_, spread or deposited annually across nearly 364 million ha of farmland or discharged in local waters, present enormous environmental challenges for the US.

The challenges are amplified when other sources of N and P not considered herein are taken into account. This analysis has conservatively estimated the wastes from CAFOs, as not only the total number of such operations is likely underestimated as noted above, but the waste from many small CAFOs remain un- or under-counted or un-permitted. Several other major pathways of nutrient inputs from the food system were also not addressed here. Meat packing plants, often located near CAFOs and owned by the same companies, contribute substantially to nutrient pollution derived from the blood, urine, feces, fat and meat tissues that are flushed in wastewater streams, yielding high levels of nitrates and other N forms (e.g., Kundu et al. 2013). Moreover, greenhouse gas emissions from the fertilizer industry itself were not included in the analysis herein. Most of the fertilizer produced in the US is either NH_3_ or urea, both of which require natural gas and both of which emit CH_4_ (Zhou et al. 2019). Although small relative to other sources, CH_4_ emissions from this source are estimated to be many-fold higher than the values formally reported from this source (Zhou et al. 2019).

The estimates reported here also have large inherent variability. Many of the conversion factors applied herein have large associated errors. Sales data for fertilizer may not be an accurate reflection of use on specific lands (e.g., Fixen et al. 2015), animal manure conversion factors are changing and fertilizer use efficiencies are improving in some areas (Yang et al., 2016; Sabo et al. 2019). Many farms are also better managed than others. Individual farmers may be applying too little or too much fertilizer or manure, and use efficiencies vary greatly with soil type, moisture, temperature, timing of application, and a myriad of other factors. Practices also vary widely with respect to manure management, including the rate and method by which it is applied to land and environmental conditions at the time of application. Nevertheless, the overarching trends reported here in time and space tell a compelling story of how nutrient pollution is changing and how crop, animal production, and human populations are generally contributing to this pollution throughout the US.

Key trends are that N fertilizer use is increasing relative to that of P, leading to an increase in N:P proportions of total inputs, N fertilizer use exceeds that of manure N inputs, while fertilizer P inputs are more comparable to manure P inputs. Fertilizer P use has been declining in part due to the accumulation of residual P fertilizer in soils over time (e.g., Zhang et al. 2017; Bouwman et al. 2017). Emissions of NH_3_, while lower than those of fertilizer input, can be regionally high (even when conservatively estimated), with livestock contributing more than fertilizer volatilization. Greenhouse gas emissions due to manure management have been rising rapidly. Overall, N and P fertilizer input and animal waste far exceeds that of people, except the densely populated northeast and southwest regions. Globally, the ratio of animal feces to human feces has been estimated to be ~ 5 in 2014 and is projected to increase to 6 by 2030 (Berendes et al. 2018). A previous analysis reported that livestock in the US produces 3 times more waste than the US population (US EPA 2003). A similar conclusion was reached by Sabo et al. (2019) for N. Even though total inputs of human waste are less than inputs of fertilizer and manure, the current (2012) estimate is that 45% of municipal wastewater is discharged directly into surface water in the US (Ivahnenko 2017), so this source can be regionally significant.

There have been multiple efforts in recent years to characterize and inventory the N and P budgets at the US national scale, or at a more detailed spatially-explicit level (e.g., Ruddy et al. 2006; Sobota et al. 2015; Houlton et al. 2013; Bouwman et al. 2017; Swaney et al. 2018a, b; Sabo et al. 2019). Ruddy et al. (2006) reported farm and non-farm fertilizer use, livestock manure by animal type and atmospheric deposition for each US county for the years 1982–2001. Yang et al. (2016) examined trends in livestock manure in the US from 1930 to 2012. Swaney et al. (2018a, b) applied the Net Anthropogenic Nitrogen Input model for the US, and more recently, Sabo et al. (2019) reported for each hydrological unit of the US, the N inventories for 2002, 2007 and 2012. The Sabo et al. (2019) approach took into account a comprehensive suite of factors, including human waste, agricultural fertilizer use, and manure production reported here, as well as partial N use efficiency on agricultural lands, N_2_ -fixation, lightning, forest fire emissions, fossil fuel combustion, among other factors to derive total N surpluses. Over this time, increased agricultural fertilizer and manure inputs offset estimated reductions in total atmospheric N deposition (Sabo et al. 2019). A similar inventory approach for each hydrologic unit of the US was determined for P (10.23719/1504278). Global analyses of N and P from agriculture and livestock production have highlighted similar trends (e.g., Bouwman et al. 2013, 2017). That is, N inputs are increasing faster than those of P, they are emitted to the environment via air and water, and due to legacy of nutrient management in agriculture during the 1970s and 1980s, combined with recent changes in inputs, the ratio of N:P exported to fresh and marine waters has increased markedly (Elser et al. 2009; Glibert et al. 2014; Beusen et al. 2015, 2016; Bouwman et al. 2017).

A recent assessment of NH_3_ atmospheric concentrations based on passive samplers across the US reported that concentrations have increased over the past decade (Butler et al. 2016). This trend is in spite of the data suggesting little of no trend in NH_3_ emissions. The explanation in these contradictory trends may lie in the decline of NO_X_ and SO_2_ emissions, providing less substrate for particulates to form, allowing concentrations of NH_3_ to increase even if emissions have not (Butler et al. 2016). Emissions of NH_3_ are conservatively estimated here for the most recent animal inventories, using published emission factors (Bowen and Valiela 2001). Estimates of emissions of NH_3_ from agricultural system have considerable uncertainty (Beusen et al. 2008), and there are several reasons why new emission factors have been proposed (Pelton et al. 2020). Much larger birds are being grown compared to 15–20 years ago; older estimates are based on European practices of litter management within the flocks and US practices yield twice the NH_3_ emission per broiler barn than comparable European barns. Thus, the likely contribution by broilers to NH_3_ emissions is a higher percentage relative to other animal sectors and the overall total could be much higher (Fig. 13c).

### Eutrophication and algal blooms

Hypoxia and HABs due to eutrophication are increasing in frequency and magnitude in both fresh and marine waters (e.g., Anderson et al., 2002, Heisler et al. 2008; Glibert et al. 2005, 2006, 2014; Glibert and Burkholder 2018). Compared to the 12 million MT of N fertilizer used in the US, it is estimated that 1.15 million MT (or about 10%) of N flows into the Gulf of Mexico annually (von Reusner 2019) contributing to the hypoxia there. The Gulf of Mexico is a prime example of how eutrophication problems can be spatially and temporally displaced from the original nutrient source (Conley et al. 2009; Paerl 2009; Glibert et al. 2011; Glibert 2020). Aside from the nuisance they cause, HAB toxins contaminate drinking water supplies, as was the case in Toledo in 2014 when 500,000 residents were told not to use tap water due to microcystin contamination (e.g., Fitzsimmons 2014), and in coastal waters, HABs also contaminate seafood supplies, cause fish kills, and, depending on species, respiratory distress among many other human and ecosystem health effects (e.g., Landsberg 2002; Backer and McGillicuddy 2006; Basti et al. 2018, Gratton et al. 2018 and references therein).

**Fig. 18:**
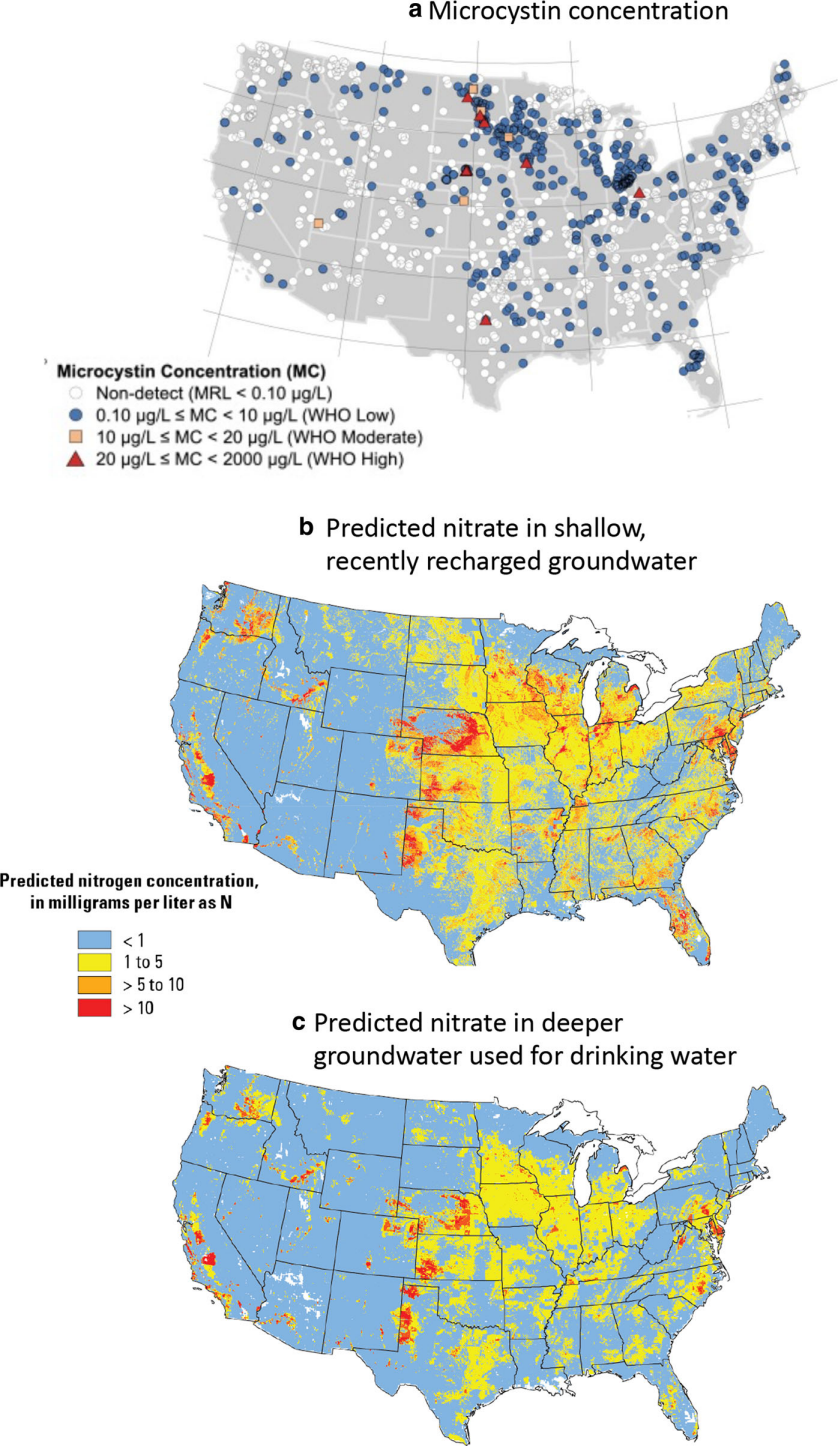
Maps of **a** concentrations of microcystins in US lakes, **b** predicted NO_3_ in shallow, recently recharged groundwater, and c that of deeper groundwater used for drinking water. Panel a reproduced from Loftin et al. (2016) with permission from Elsevier. Panels **b**, **c** reproduced from USGS (https://www.usgs.gov/media/images/predicted-concentrations-nitrate-us-groundwater; public domain)

Control of P has been long been promoted to curtail freshwater HABs because it is easier to control than N, and has long been considered the limiting nutrient in freshwaters (e.g., Schindler et al. 2008, 2016, Schindler and Hecky 2008). It has also been long been thought that if N is reduced well below balanced proportions, there can be growth of N_2_-fixing cyanobacteria among which are toxic species and they will compensate for N limitation by accessing the atmospheric source (e.g., Schindler et al. 2008, 2016 and references therein). Thus, it would seem that the trend in increasing N:P should be viewed positively. However, the trend of increasing N:P proportions in fertilizer inputs is particularly concerning for several reasons. Many HAB cyanobacteria are not N_2_-fixing, for example, *Microcystis*, and their occurrences are increasing in freshwaters around the world in direct proportion to increasing N loads (Glibert et al. 2014 and references therein). *Microcystis* is increasing throughout the US, but the Midwest is a hot spot for blooms—and for more toxic blooms—due to agricultural impacts (Fig. 18c; Michelak et al. 2013; Loftin et al. 2016). Many marine and estuarine dinoflagellate HABs also have been shown to be more abundant under conditions of increasing N:P. Examples of high biomass HAB dinoflagellates occurrences in environments where N:P loads are in excess of Redfield proportions can be found in the Baltic Sea (Hajdu et al. 2005), Delaware Inland Bay (Handy et al. 2008), Neuse River Estuary (Springer et al. 2005), Chesapeake Bay (Li et al. 2015) and East China Sea (Li et al. 2009; Glibert et al. 2014) among many other regions.

The second problem with a focus on P control over N control is that many cyanobacteria and marine or estuarine dinoflagellate HABs (among other HAB taxa) may be, in fact, more toxic when N is in stoichiometric excess over P. Thus, contrary to the concern that N limitation will promote toxic cyanobacteria, the toxicity of many HABs increases as N:P increases (Glibert 2017 and references therein). Most notably, excess N over P availability has been related to microcystin production under controlled chemostat conditions and in natural populations (Oh et al. 2000; Van de Waal et al., 2009; Harris et al. 2016). In the dinoflagellate *Alexandrium tamarense,* saxitoxin production increased by three- to fourfold under P deficiency (Boyer et al. 1987; Guisande et al. 2002, reviewed by Granéli 2005; Granéli and Flynn 2006), and toxicity of the dinoflagellate *Karlodinium veneficum* increased under P limitation, but especially in combination with elevated levels of CO_2_ (Fu et al. 2010). Similarly, toxin production by the dinoflagellates *Gymnodinium catenatum, Alexandrium excavatum* and the diatom *Pseudo-nitzschia multiseries* also increased under P stress (Granéli and Flynn, 2006). Many toxins are rich in N and accordingly N-rich toxins can accumulate in excess under P limitation (e.g., Granéli and Flynn 2006; Van der Waal et al. 2014 and references therein).

Adding to the trends of increasing N relative to P are the atmospheric NH_3_ emissions from animal feeding operations. Most such emissions are deposited within 2.5 km of the source, based on studies of emission from broiler houses on the US eastern seaboard (Baker et al. 2020). These emissions, derived from the animal houses themselves, manure handling, or land applications, have multiple environmental effects. Not only do these emissions contribute to eutrophication (e.g., Mallin and Cahoon 2003; Galloway et al. 2014), but they can form fine aerosols as NH_3_ is converted to NH_4_ and deposited on particles, contributing to haze, impaired visibility and respiratory problems. These aerosols can also be deposited as NH_3_/NH_4_ on nearby forests or crops which can, in turn, elicit stress responses from acute NH_3_/NH_4_ exposure (e.g., Fangmeier et al. 1994). Recent modeling has shown that there has been a threefold increase in soluble N deposition over land and a twofold increase over the ocean due to human activities (Kanakidou et al. 2016), driven largely by NH_3_ emissions from agriculture that have traveled from the original source.

These trends all underscore that nutrient reduction efforts must focus on both N and P, even for regional systems that are classically considered to be “limited” by one nutrient or the other (e.g., Burkholder et al. 2006; Howarth and Paerl 2008; Conley et al. 2009; Paerl 2009; Glibert et al. 2011, 2013; Glibert 2017, 2020). Dual nutrient strategies, however difficult they are to achieve, should be the goal, as multiple ecological and ecoservice benefits are met by reducing N input (Vitousek et al. 1997) even in classically P-limited systems, such as lakes. Fragmenting sustainability arguments and focusing on single nutrient reduction measures undermines the need to protect multiple ecosystem services at broad spatial scales, especially given that many eutrophication problems are displaced from the original nutrient source, as previously described for the Gulf of Mexico.

To promote a more environmentally-favorable image, the fertilizer industry has been advocating that farmers apply the “4R” rule for fertilizers: the right source at the right rate, right time and right place (https://www.nutrientstewardship.com/4rs/). This same right-place-right-time principle applies to the kinds of algal species that respond in receiving waters of these wastes. It takes the right nutrients at the right time relative to the needs of the primary producers (algae) for blooms to form (Glibert and Burford 2017). While over-enrichment of both fresh and coastal waters by nutrients is a major pollution problem worldwide, it is not only total nutrient loads that promote HABs and alter microbial biodiversity, it is the right nutrients at the right time.

Many HAB taxa also appear to be favored over diatoms when N is supplied in chemically-reduced relative to oxidized forms—as, for example, in the form of urea (Glibert et al. 2006, 2014). The shift toward increasing use of urea stems from several advantages it has over other N forms (Glibert et al. 2006). It is less explosive than NH_4_ and NO_3_ when stored, and it can be applied as a liquid or solid. The increase in global use of urea has been related to HAB increases (Glibert et al. 2006, 2014, 2016), and similar conclusions can be drawn for various parts of the US where urea use has increased. For example, cyanobacterial blooms in Florida Bay and on the southwest Florida shelf have been shown to be positively correlated with the fraction of N taken up as urea, and negatively correlated with the fraction of N taken up as NO_3_^−^ (Glibert et al. 2004). Use of slow-release fertilizers has been promoted to reduce leaching of N; slow release fertilizers are coated urea-based granules that may contain a urease inhibitor. The use of urease inhibitors delays the hydrolysis of urea for up to several weeks and thus increases the likelihood that runoff or overland transport will contain urea and not its decomposition products (Prakash et al. 1999). Use of slow-release fertilizers may help to reduce hydrolysis in the soil, but may contribute to runoff of forms of N that are more favorable for at least some HABs.

Recently another environmental consequence of algal blooms has been reported: that is, blooms are an important contributor to CH_4_ emissions (Beaulieu et al. 2019 and references therein; Fig. 1b). Production of CH_4_ in lakes and eutrophic impoundments is directly related to the chlorophyll *a* concentration of the water (DelSontro et al. 2018). Beaulieu et al. (2019) estimate that CH_4_ emissions from eutrophic lakes will increase 30–90% over the next century due to continuing eutrophication pressures. Moreover, these authors reported that an increase in P loading by 1.5 times will lead to CH_4_ emissions that are equivalent to that from wetlands, currently the largest single source. The continued nutrient pollution from crop and animal production clearly multiplies the impact on greenhouse gases due to accumulations of algal biomass and its decay. It is now abundantly clear that the historic view of algal responses to eutrophication—i.e., that increased nutrients promote increased chlorophyll and high-biomass blooms leading to oxygen deduction and losses in habitat (e.g. Cloern 2001)—is far too simplistic for understanding how harmful taxa develop in response to changes in nutrients.

### Human health and community impacts

Numerous studies have documented the many human health impacts of populations living in the shadow of large animal operations. Casey et al. (2015) reviewed the literature of the past 2 decades and reported that four types of health problems were consistent related to life near CAFOs: respiratory issues, methicillin-resistant *Staphylococcus aureus* (MIRS), Q fever (caused by the bacteria *Coxiella burnetii* typically transmitted from animals), and mental health (stress). Occupational-related asthma and bronchitis is not unusual among farm workers or family members, nor is exposure to dangerously high concentrations of NO_3_ in drinking water, especially given the fact that many rural areas draw water from local wells rather than municipal supplies (reviewed by Burkholder et al. 2007; Miller and Muren 2019; Fig. 18b,c). High concentrations of NO_3_ in water supplies have been associated with increased risks of blue baby syndrome, some cancers (including colon, kidney, stomach, ovarian and bladder), reproductive effects, and diabetes (reviewed by Burkholder et al. 2007; Casey et al. 2015; Miller and Muren 2019). Other contaminants in water near CAFO-impacted communities include veterinary antibiotics or hormones, pesticides, and other pharmaceuticals seep into surface and groundwater from applications to sprayfields or leak from poorly constructed or aging lagoons (Burkholder et al. 2007 and references therein).

Emissions of NH_3_ from CAFOs can trigger asthma attacks. Often emissions of H_2_S co-occur with NH_3_ emissions, especially from poultry houses. It has been reported that people frequently exposed to these emissions were 66% more likely to be diagnosed with pneumonia (Poulsen et al. 2018).

Substantial amounts of fecal bacteria remain in manure when this material is spread on land. While many such microbes may be killed by exposure to ultraviolet radiation (Crane et al. 1983), many remain viable. Viability can be maintained when these materials enter groundwater or surface waters (Mallin and Cahoon 2003). Burkholder et al. (1997) observed that fecal bacteria could be found in river waters and sediments months after a large swine waste spill, but even without large spills, chronic exposure can be problematic.

### Economics and trade-offs

Ewing and Runck (2015) modeled the trade-off between the need to optimize high rates of N fertilization of corn and the cost of water quality impacts in the Midwestern US—and highlighted the “deep conflict” between stakeholders involved in food production and those using water resources. Their analysis underscored the importance of understanding regional (less than county level) variabilities where optimizations can be gained and emphasized the importance of stakeholder involvement at local scales. They showed that technological solutions do exist that could increase corn production and improve water quality. Yet, Herrero et al. (2015) argue that even with the efforts over the past decade to quantify impacts of the “gargantuan appetite for livestock products”, integrating these efforts with economic and sociocultural efforts is seldom done, when climate, nutrient cycles, biodiversity, land degradation, deforestation are collectively considered.

**Fig. 19:**
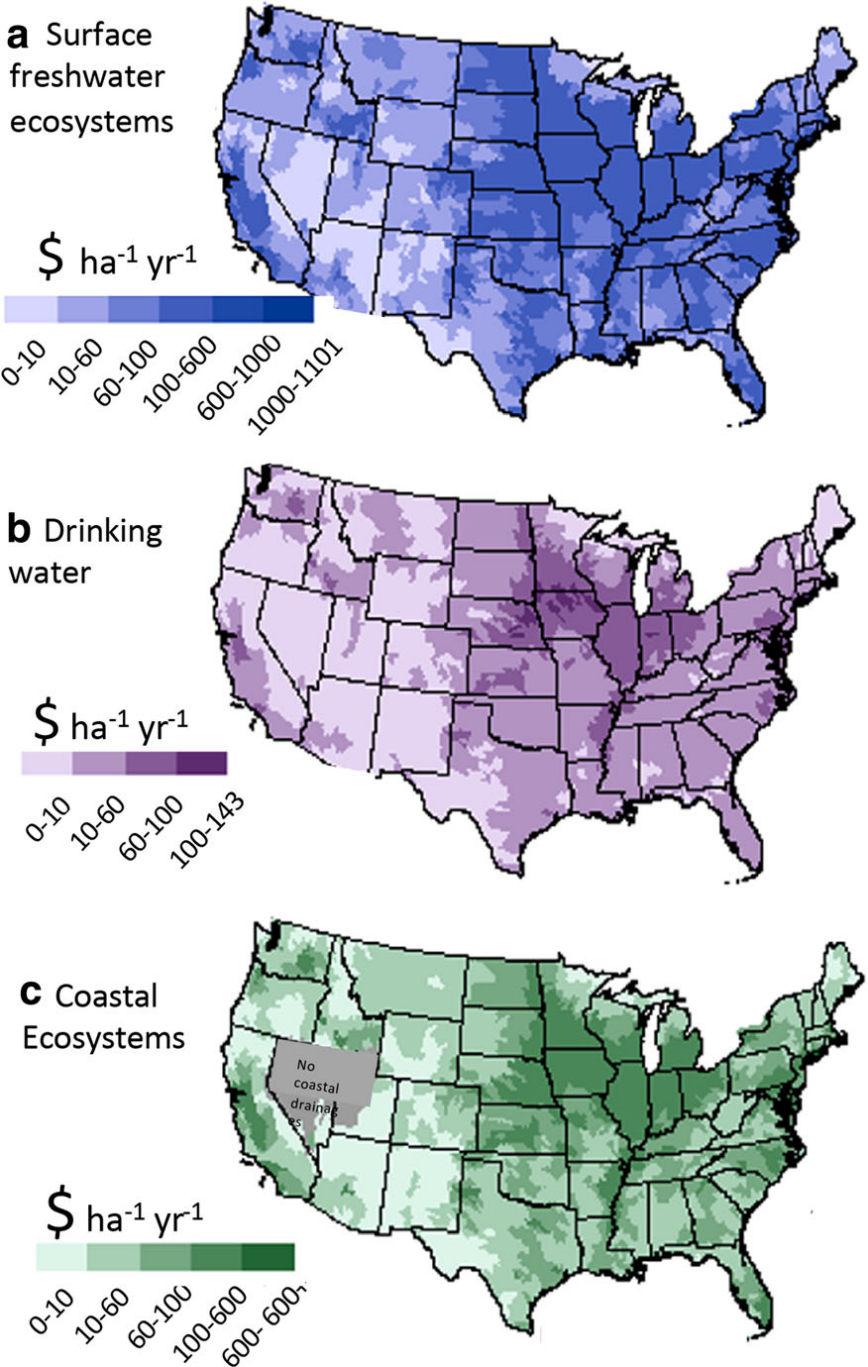
Estimated costs of N pollution in the US on **a** surface freshwater ecosystems, **b** drinking water, and **c** coastal ecosystems. Reproduced from Sobota et al. (2015) under Creative Commons 3.0 license

Costs to reduce and mitigate nutrient pollution are extremely high. A recent estimate from USDA (cited in Ribaudo et al. 2011) suggests that $2 billion annually is spent removing NO_3_ that originates with cropland applications and that two-thirds of US cropland is not meeting criteria for good N management. Sobota et al. (2015) estimated the economic costs associated with the leakage of N from the production of food, fuel and fiber in the US. They calculated the damage cost in mitigation, remediation, direct damage or substitution for each N source (focusing on synthetic fertilizers) and human health and environmental impacts by applying methodology described by Birch et al. (2011, Compton et al. (2011) and van Grinsven et al. (2013). They estimated that in the year 2000, the damage costs for N leakage ranged from $1.94 to $2,255 ha^−1^ for different hydrological zones as defined by the USGS. Of these damages, 73–77% were associated with leakage of agricultural N, and areas with the largest damage to aquatic habitat and eutrophication were in the upper Midwest and central California (Fig. 19). Interestingly, they also calculated that much of mid Atlantic, Pacific Northwest, as well as southern California, received less N annually than the Midwest yet had similar damage costs because of the high costs of air pollution on human health. Across the nation, they estimated that damages ranged from $19 billion associated with drinking water impacts to $78 billion associated with freshwater ecosystems, and overall the median estimates in all damages was $210 billion in the early 2000s. This figure represent 21% of the estimated $992 billion that the food and agriculture industry contributes to the US economy (as of 2015; https://www.agweb.com/article/food--ag-industry-contributes-992-billion-to-us-economy-NAA-ben-potter). NOAA published a similar finding, estimating that $82 billion was lost each year in lost fishing revenues and human health problems associated with algal blooms (https://aamboceanservice.blob.core.windows.net/oceanservice-prod/ecoforecasting/noaa-ecoforecasting.pdf). Yet, in keeping with Herrero et al.’s (2015) central point that the economic and societal costs of livestock production must be better understood, undoubtedly, the economic impacts estimated by Sobota et al. (2015) would be higher today and would be higher if the damage from leaked N from the increasing number of animal operations were also considered. A very recent report estimates that the total hidden costs of the food industry across the world to be in range of $12 trillion yr^−1^, accounting for water scarcity resulting from agriculture use, biodiversity loss and greenhouse gas emissions–a value approaching the domestic product of China (Nature 2019).

### Threats to current and future farming

Farming practices will evolve, whether or not such changes will be driven by sound policies, or factors beyond regulatory control. The consolidated, and seemingly highly efficient, food production system is not resistant to change. Its fragility, in both the short term and long term is evident.

The current tariffs on soybean and pork imposed by the Chinese on US exports clearly affect production in the short term. Farmers are being squeezed by these policies in many different ways. Many farmers are going bankrupt. On average, 7 dairy farms went bankrupt per day in 2018 (https://www.farmpolicyfacts.org/2019/04/our-view-trade-can-kickstart-ailing-farm-economy/). Bankruptcies have increased in 9 of the 10 regions of the country; in Regions IV, V, VI and VII, these numbers totaled 81,125, 62 and 87 in 2018 (Wilton and Newton 2019). These bankruptcies are mostly those of the remaining small farmers; large corporations have more capital to buffer these downturns. Subsidies have aided farmers especially in the upper Midwest (Regions VII and V), but have disproportionately aided the industrial farming conglomerates. Consolidation of large farms will only increase.

At the time of this writing, there has been a rapid acceleration in the rate of burning of the Amazon rainforest (Sullivan 2019; Ortiz 2019). The number of fires is about 35% higher than in the first half of the year for all years since 2010, and has risen 79% since 2018 (Ortiz 2019). These fires have been largely deliberately set to clear forests for the planting of soybeans and for cattle grazing. If the US is no longer the world’s breadbasket, other countries will take this role. Brazil has currently replaced the US as the major provider of soybeans for China, and as soybean production in Brazil has ratcheted upwards, it is becoming well positioned to become the world’s leading supplier (Sullivan 2019). Thus, Brazil burns to create new farmland from the Amazon as small US farmers struggle, both in response to changes in US-China trade relationships, with large international, industrial farms able to capitalize on both of these changes.

One recent factor that industrial farms have not been able to control is the impact of the global 2020 coronavirus pandemic. Many US meat packing plants closed for periods of time due to employee illness. Consequently, many hogs and broilers were euthanized, placing more economic hardships on US producers. These carcasses are being disposed in landfills or composted for fertilizer (Pitt 2020). The full impacts of trade tariffs, the pandemic and other short-term pressures are yet to be seen, and future inventories at local and regional levels will tell those stories.

In the longer term, it is projected that P reserves may be exhausted in a few decades (e.g., Daneshgar et al. 2018). The demand for N, however, is estimated to continue to escalate. For North America, the rate of N use may increase by 32% and that of P use by 24% relative to 2005, based on estimates of Drescher et al. (2011). Globally, urea use is projected to double by mid-century (Millenium Ecosystem Assessment 2005, Glibert et al. 2014 and references therein). This will continue to drive the N:P of runoff higher, with environmental consequences downstream.

The United Nations recently released a report on climate change (IPCC 2019) which details how interactions between climate change, greenhouse gas fluxes, extreme events (floods and droughts), land use change, and desertification may threaten food and nutritional security. Temperatures and CO_2_ are rising–factors that may seem beneficial for the growth of some crops. Favorable regions for certain crops may migrate. There is some evidence that higher temperatures are favoring corn production in Minnesota, but disfavoring yields in Illinois, Indiana, and Ohio, and also favoring soybean production in the upper Midwest while disfavoring wheat (Belz 2019). Extreme heat can also alter the timing or rate of flowering, in some cases rendering plants sterile (Dukes and Hertel 2018). Disease and pests may change in frequency. Increased temperatures also reduce the feeding rate by animals and increase their susceptibility to disease.

Under changing climate, precipitation is less predictable, often coming in fewer, more concentrations events. High rainfall makes planting difficult, flooding late in the season can drown plants, but too little rainfall also kills plants (Dukes and Hertel 2018). The extent to which changing precipitation patterns will affect farm production in the long run is yet to be determined. The Midwest experienced massive flooding in 2019, leading to the inability of many farmers to even sow their crops. The 2018–2019 planting season was the wettest in recent history, and the past 5 years have also experienced very wet April–May periods (https://mrcc.illinois.edu/pubs/docs/GL-2018_Climate-trends-and-impacts-summary.pdf). Accordingly, fields were left unplanted, and while this led to higher prices for corn and soybean due to reduced supply, the lack of crop to sell does not balance this loss for farmers. This flooding follows the devastating Midwest drought of 2012. As a crop highly sensitive to heat and water stress, corn is definitely at risk for future and will see more market volatility in the years to come. Recent modeling suggests that in the Midwest, water balance changes due to increased temperature and reduced snowfall may be more important than increased precipitation in the next half decade (Kalcic et al. 2019).

One approach farmers have used to overcome this risk is to forsake traditional crop rotation (corn and soybean) for continuous corn production. In 2012, 22% of corn production was in continuous rotation, a practice that leads to more fertilizer use as well as more soil erosion (Barton and Clark 2014). Moreover, some corn hybrids are becoming more sensitive to drought, requiring higher rates of irrigation during drought periods (Barton and Clark 2014).

Intensive precipitation also leads to greater runoff of both fertilizer and of soil itself. Yet, precipitation events may affect N and P differently. On the one hand, P, which is often bound to particles can be more easily transported by overland flow, whereas N, especially as NO_3_, more readily leaches into the ground and may or may not be mobilized to adjacent waters (e.g., Sims et al. 1998). In situ time series of nutrient monitoring in tributaries of the Chesapeake Bay confirmed these different patterns following rainfall events (Glibert et al. 2005). On the other hand, the accumulation of P in soils over time contributes to retention of P relative to N, and a further skewing of the N:P ratio in exported nutrients (Beusen et al. 2016; Bouwman et al. 2017).

Climate changes also pose other risks. There is now considerable emerging evidence that in a higher CO_2_ environment, the nutritional quality of plants, including the cellular content of N, protein, and vitamins, is reduced, especially for those plants having C3 metabolism (e.g., rice, wheat) (Loladze 2014; Weigel 2014). This, in turn, may alter the food quality for the animals that are dependent on those crops and may contribute to negative shifts in human nutrition as well. Large, industrialized operations are far less nimble in their ability to adapt to change than smaller operations.

### Opportunities and impediments for advancement

Numerous scientists have suggested approaches that can be undertaken globally to mitigate nutrient pollution (e.g., Sutton et al. 2013; Conant et al. 2013; Billen et al. 2015; Bouwman et al. 2017). In the US, legislative efforts related to nutrient pollution from farms are not advancing in the right direction. The Farm Bills of recent years have cut the conservation provisions considerably which were originally included in the 1985 Farm Bill. Moreover, funds available through the Environmental Quality Incentive Program in the 2002 Farm Bill, meant to incentivize farmers to idle lands and to implement other environmental improvements, were allowed to be used for the construction of manure lagoons (Imhoff 2019). Further degradation of waters may result from the current administration’s efforts to roll back the definition of “waters of the United States” under the Clean Water Act, thus releasing regulations on many wetlands and tributaries that were protected since 1986 and which was broadly enforced by the EPA since 2015 (Eilperin and Dennis 2019). Wetlands and tributaries are often first recipients of farm runoff.

It is unlikely that the economic and policy drivers favoring large agricultural systems will change any time soon. Much has been written about best management practices, fertilizer use efficiency and potentials for improvement (e.g., Bouwman et al. 2009; Fixen et al. 2015; Mueller et al. 2017; Zhang 2017; Clark and Tilman 2017; Alexander et al. 2017). Davis et al. (2015) modeled the global impacts of livestock intensification, and specifically the shift to dependence on grain. They found that animal calories produced from feed were more efficient than those produced from non-feed sources in terms of land use and greenhouse gas emissions, but conversely production from feed required substantially more N per animal in the overall production chain. Livestock fed poorer quality feed produce more CH_4_ than those fed forages that are more nutritious (https://extention2.missouri.edu/g310). Others have suggested other approaches that can be taken to reduce nutrient pollution, such as reduction of food waste and improved processes for mitigating or removing N pollution from the environment (e.g., Houlton et al. 2019 and references therein). While major improvements in use efficiency can be implemented in parts of the world where fertilizer use is less fine-tuned to specific crops and soil types, it is unlikely to ever reach a point where there is no environmental loss. The difficulty in improving efficiency of N use particularly lies in the high mobility of N in the soil–plant system, and the variety of potential loss pathways, ranging from volatilization of NH_4_^+^, denitrification, leaching and runoff and other pathways (Bouwman et al. 2009). While both P and N have been accumulating in soils (e.g., Van Meter 2016, 2017, Zhang et al. 2017), leading to opportunities for fertilizer reductions, sales of N relative to P fertilizer continue to rise.

**Fig. 20:**
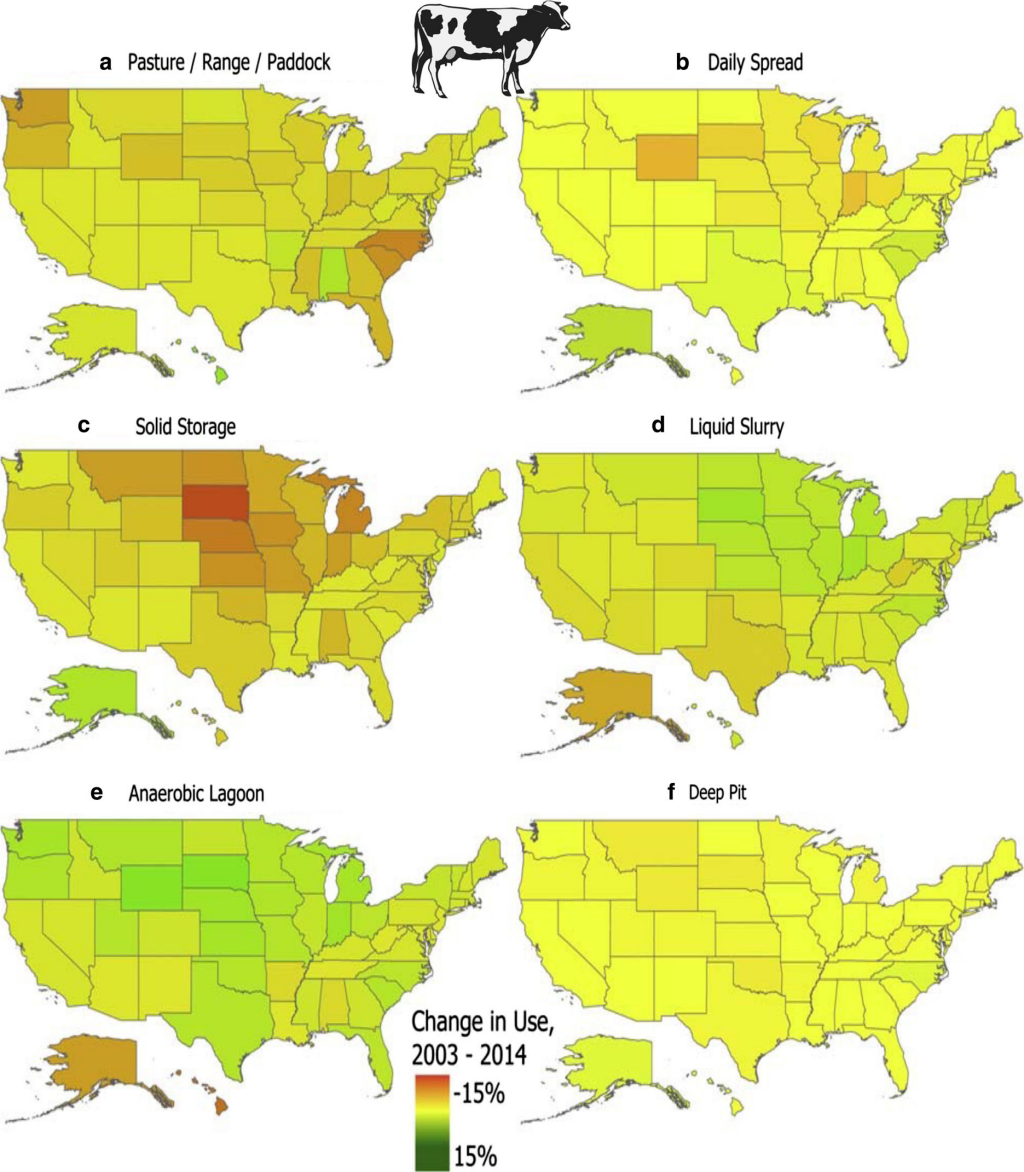
Change in different waste management strategies of dairy operations in the US from 2003 to 2014 Reproduced and modified from Niels and Wiltshire (2019) under Creative Commons 3.0 license

Manure management varies by animal operation and by state and there has been a shift toward liquid waste management in both the dairy and swine industries. Anaerobic lagoons and liquid slurry operations (Online Resources Table S3) are most common in dairy and hog operations (e.g., Hunt et al. 2019, Niles and Wiltshire 2019 and references therein; Fig. 20). Managing liquid manures appears to be among the “lowest hanging fruit” of nutrient control in much of the country. Manure spreading should be held to the same strict “4Rs” accounting as chemical fertilizer applications. The lagoons themselves need to be carefully managed. Lagoons, which may be clay or plastic lined, may lose integrity with age (Barth et al. 2004), leading to increased leakage. Many older lagoons are unlined. Volatilization also depends on how farmers manage their lagoons with respect to C content; NH_3_ emissions can be reduced if C-rich bedding material is used (Barth et al. 2004). Emissions vary with the bacterial content of the lagoons, especially purple sulfate bacteria (Leytem et al. 2017). Emissions also increase with temperature and pH of the holding lagoon (Arogo et al. 2003; Harper et al. 2004; Doorn et al. 2002; Leytem et al. 2017; Peterson 2018 and references therein). Emissions are also highly variable with short-term wind and precipitation events, with increases in CH_4_ emissions from dairy lagoons during rainy days (Grant and Boehm 2015; Leytem et al. 2017). Covers may help to limit these emissions. There are some efforts to use pig manure and corn silage for biogas production (e.g., Gaworski et al. 2017). This technology is beginning to be applied in North Carolina, where Smithfield Foods, now a Chinese company, has partnered with Duke Energy (e.g., Coker 2018). Ultimately, waste treatment may become the only mechanism by which real nutrient reductions can occur. If water quality is valued and if the costs of algal blooms and other environmental impacts are fully recognized, wastewater treatment for animal operations may eventually become economically sound.

Some practices or policies appear to provide favorable environmental outcomes but there can also be unintended consequences. Organic farming, for example, may reduce use of some chemical fertilizers, but this reduction in fertilizer use creates another problem: yields are lower, by as much as 8–25% (Baldos 2018). Therefore, organic farmers have to convert more lands to agricultural fields to produce the same quantity. Moreover, organic nutrients, which favor the growth of many types of HABs, are used to a greater extent in organic farming, leading to increased leakage of these forms to local waters. Weed control on organic farms also requires more mechanical cultivators, leading to more soil erosion and other associated secondary problems (Gunderson et al. 2018). As another example, some animal operations are moving to cage-free operations, particularly in the poultry industry where there is pressure for more humanely-raised products. Many restaurants, including McDonald’s, are committed to using eggs only from cage-free systems. Yet, these systems lead to higher NH_3_ emissions and other air quality problems due to the greater accumulation of manure and scratching that the birds do while exposed to this dust and litter (Erickson 2018). These changes could have large regional impacts, as chicken producers in the mid-Atlantic (Maryland and Delaware) currently contribute about 17% of the N load of Chesapeake Bay (https://cen.acs.org/environment/pollution/Livestock-emissions-still-air/96/i14). There are no simple solutions that will unravel the profitability and environmental impacts from large agrobusinesses–especially in the current US policy climate.

By definition, CAFO lagoons are “point sources” of pollution and, depending on the size of operation and waste handling procedures, must be permitted under the Clean Water Act, which requires operators to have a nutrient management plan and which defines the limits on the allowable amount of discharge to local waters. Such regulations have been regularly revised (US EPA 2010) and regularly challenged in court. As noted above, state-wide reporting–and therefore the transparency of state-wide statistics–of CAFOs is low for almost every state (Miller and Muren 2019). Permitting can be avoided if the size of the operation falls just under the regulatory limit, and the percentage of CAFOs reporting permits to the EPA (https://www.epa.gov/sites/production/files/2019-09/documents/cafo_tracksum_endyear_2018.pdf) is astonishingly low, especially for those states where hog production is high (Fig. 11b; Online Resources Fig. S6c). Permitting can also be avoided if the facility does not discharge directly to a waterway. Lack of permitting does not imply illegal operation, only that the configuration (i.e., number of confined animals or waste management procedures) of the farm differs from that required to be regulated. The animals from unpermitted operations nevertheless still release nutrients. Moreover, federal inspections and enforcement of CAFOs have declined every year since 2011; in 2016, enforcement actions were down 75% and inspections down more than 50% compared to those actions taken during the Obama administration (Walton 2016).

There are no federal policies as of yet regarding the emissions of CH_4_ or N_2_O from CAFO operations (Tomas 2019), nor is this a politically favorable time to suggest new policies or regulations. Because farmers and ranchers are exempted from reporting emissions to federal agencies, the US EPA methodology for estimating emissions is under continual evolution. This exemption from reporting was reaffirmed in the recent Farm Act (Erickson 2018). As seen from the permitting percentages, most farming waste disposal does not fall under the Clean Water Act, but it has been suggested that as emitters of greenhouse gases, farm operations, and especially CAFOs, could, however, fall under some previsions of the Clean Air Act (Tomas 2019). Others (e.g., Ruhl 2000) have argued that the “geographic, economic, and political settings of the farming industry call for approaches that may be outside the box of conventional environmental law. The environmental regulation of farms must incorporate several key features if it is to succeed where traditional models of environmental law surely would not”. Such an approach would balance environmental regulation with tax incentives and trading programs. As noted above, it is unlikely that such a sweeping new approach to environmental regulation of farming will happen any time soon.

## Conclusions

This paper has attempted a broad review of the patterns and trends in nutrient inputs and greenhouse gas pollution arising from US farming practices. This analysis builds on publicly available and published data and makes use of available detailed inventories. Collectively these efforts have shown that for the entire US: (1) use of N fertilizer is increasing faster than that of P, leading to an increase in the N:P of this source; (2) fertilizer N inputs exceed those of manure, while fertilizer P inputs and those of manure are more comparable; (3) the number of CAFOs has increased over the past decades, including a near 10% increase since 2012, driven largely by a 13% increase in hog production; (4) atmospheric NH_3_ release and human wastewater total inputs are less than those of fertilizer and manure, but large regional differences exist across the country (and atmospheric NH_3_ may be underestimated); (5) while CH_4_ emissions from enteric fermentation remain the largest contributor of this greenhouse gas pollutant, CH_4_ and N_2_O emissions from manure management are rapidly rising.

At the broad scale, the industrialization of farming, driven by economics rather than a sustainability ethic, will only continue to exacerbate the eutrophication of fresh and coastal waters. There has been an upward trend in N:P of all inputs, conditions that favor many HABs and/or their toxicity. Tariffs and trade disputes are contributing to the destruction of the Amazon as Brazil steps in to lead global soybean production. Together with climate threats and uncertain political trade policies, a near-term future with reductions in nutrient and greenhouse gas emissions by the US farming industry is bleak, and the negative consequences will be felt worldwide for the foreseeable future.

## Electronic supplementary material

Below is the link to the electronic supplementary material.

Supplementary file1 (DOCX 16395 kb)

## Data Availability

All data are from publicly available sources as indicted throughout the manuscript.
