# Supplementary material for: From hogs to HABs: impacts of industrial farming in the US on nitrogen and phosphorus and greenhouse gas pollution
Source: Biogeochemistry. 2020 Aug 10;150(2):139–80. doi: 10.1007/s10533-020-00691-6 (PMC7416595; doi:10.1007/s10533-020-00691-6)
Supplement: Supplementary file 1 — Supplementary file1 (DOCX 16395 kb) [file 10533_2020_691_MOESM1_ESM.docx]

**Online Resources**

**From hogs to HABs:**

**Impacts of industrial farming in the US on nitrogen and phosphorus**

**and greenhouse gas pollution**

Patricia M. Glibert

University of Maryland Center for Environmental Science

Horn Point Laboratory

PO Box 775

Cambridge MD 21613

This Online Resource material contains additional detail related to patterns and trends reported. See associated text for references cited.

**Online Resource Table 1.**

Conversion of weight of different types of animals to equivalent units (equal to 1000 lb or 453 kg) animals

| **Animal type** | **Typical weight lbs** | **Typical weight kg** | **Equiv. animal units** |
| --- | --- | --- | --- |
| Cattle | 1000 | 453 | 1.0 |
| Dairy cows | 1400 | 635 | 1.4 |
| Hogs | 250 | 113 | 0.25 |
| Broilers* | 4.5 | 2.0 | 200 |
| Turkeys | 18 | 8.2 | 55.5 |

*Note that the average size of broiler chickens in the US has grown and is now closer to 2.7 kg.

**Online Resource Table 2.**

Nitrogen and phosphorus excretion and NH_3_ emission rates by animal type. Values are

kg animal^-1^ yr^-1^

| **Process** | **Animal type** | **N** | **P** | **Reference** |
| --- | --- | --- | --- | --- |
| Excretion | Cattle | 54.75 | 19.34 | Ruddy et al. 2006 |
|  |  | 60 | 10.5 | Bouwman et al. 2017 |
|  | Dairy cows | 74.46 | 11.68 | Ruddy et al. 2006 |
|  |  | 130 | 22.7 | Bouwman et al. 2017 |
|  | Hogs | 9.86 | 4.38 | Ruddy et al. 2006 |
|  |  | 11 | 1.8 | Bouwman et al. 2017 |
|  | Broilers | 0.54 | 0.22 | Ruddy et al. 2006 |
|  |  | 0.5 | 0.1 | Bouwman et al. 2017 |
|  | Turkeys | 1.61 | 0.62 | Ruddy et al. 2006 |
|  |  |  |  |  |
| NH_3_ emissions | Cattle | 18.7 |  | Bowen and Valiela 2001 |
|  | Dairy cows | 18.7 |  | Bowen and Valiela 2001 |
|  | Hogs | 6.0 |  | Bowen and Valiela 2001 |
|  | Broilers* | 0.24 |  | Bowen and Valiela 2001 |
|  | Turkeys | 0.86 |  | Committee on Environment and Natural Resources 2000 |

*Revised emission factor estimates for larger broilers may exceed 0.41 (Pelton et al. 2020). The more conservative value was used herein.

**Online Resources Table 3.** Definitions of manure management systems based on US EPA, (http://www.epa.gov/sites/production/files/2017-022/documents/2017_all_annexes.pdf).

| **Manure management system** | **Definition (US EPA)** |
| --- | --- |
| Pasture/range/paddock | The manure from pasture and range grazing animals is allowed to lie as is, and is not managed |
| Daily spread | Manure is routinely removed from confinement facility and is applied to cropland or pasture within 24 h of excretion |
| Solid storage | Manure is stored, typically for a period of several months, in unconfined piles or stacks. Manure is able to be stacked due to the presence of a sufficient amount of bedding material or loss of moisture by evaporation |
| Liquid slurry | Manure is stored as excreted or with some minimal addition of water to facilitate handling in either tanks or earthen ponds, usually for periods less than one year |
| Anaerobic lagoon | Uncovered anaerobic lagoons are designed and operated to combine waste stabilization and storage. Lagoon supernatant is usually used to remove manure from the associated confinement facilities to the lagoon. Anaerobic lagoons are designed with varying lengths of storage (up to a year or greater), depending on the climate region, the volatile solids loading rate, and other operational factors. Anaerobic lagoons accumulate sludge over time, diminishing treatment capacity. Lagoons must be cleaned out once every 5 to 15 years, and the sludge is typically applied to agricultural lands. The water from the lagoon may be recycled as flush water or used to irrigate and fertilize fields. Lagoons are sometimes using in combination with a solids separator, typical for diary waste. Solids separators help control the buildup of nonbiodegradable material such as straw or other bedding material. |
| Deep pit | Manure is collected and stored, usually with little or no added water, typically below a slatted floor in an enclosed animal confinement facility. Storage periods range from 5 to 12 months, after which manure is removed from the pit and transferred to a treatment facility or applied to land. |


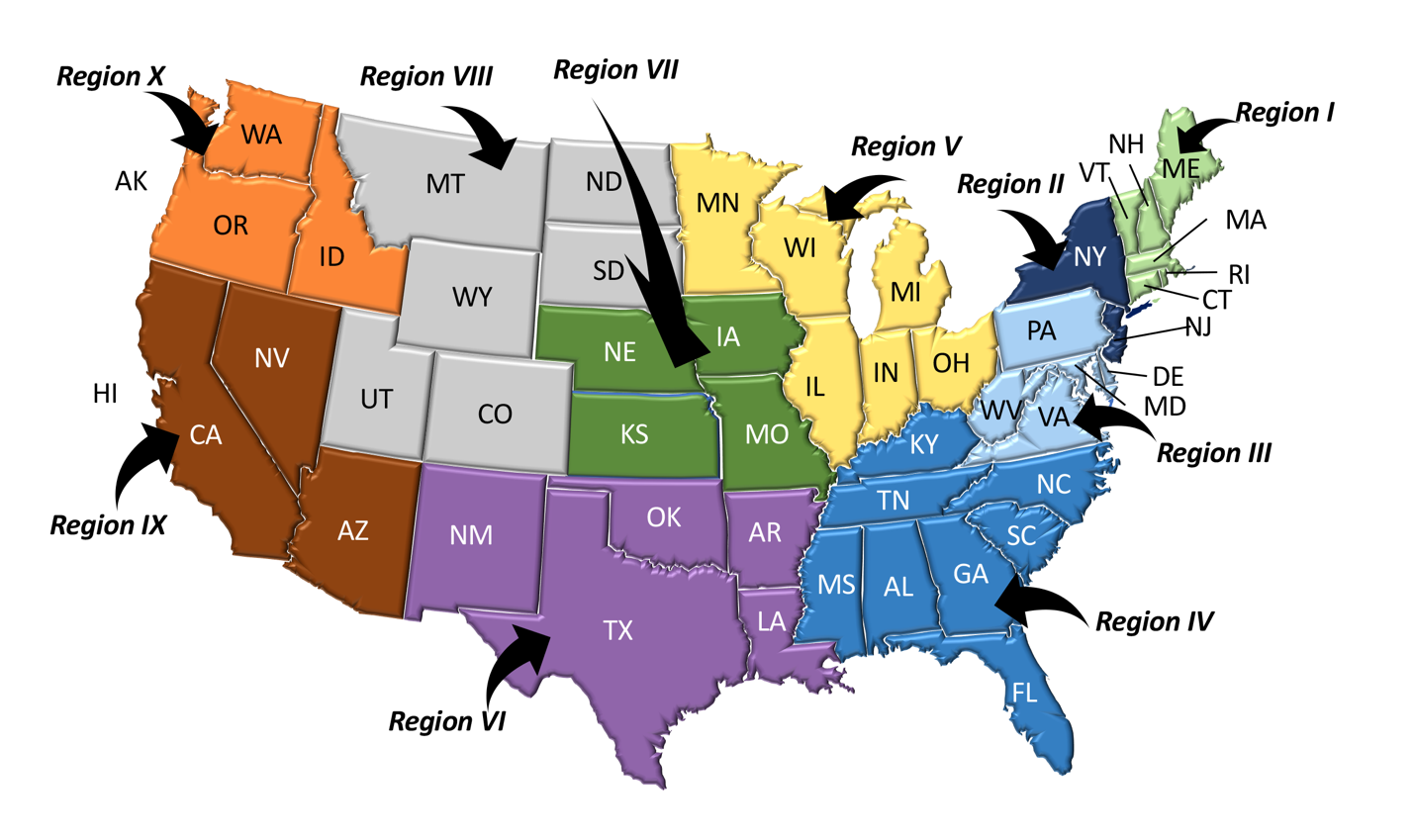
 **Online Res. Fig. 1.** The 10 regions of the US as designated by the Office of Management and Budget.


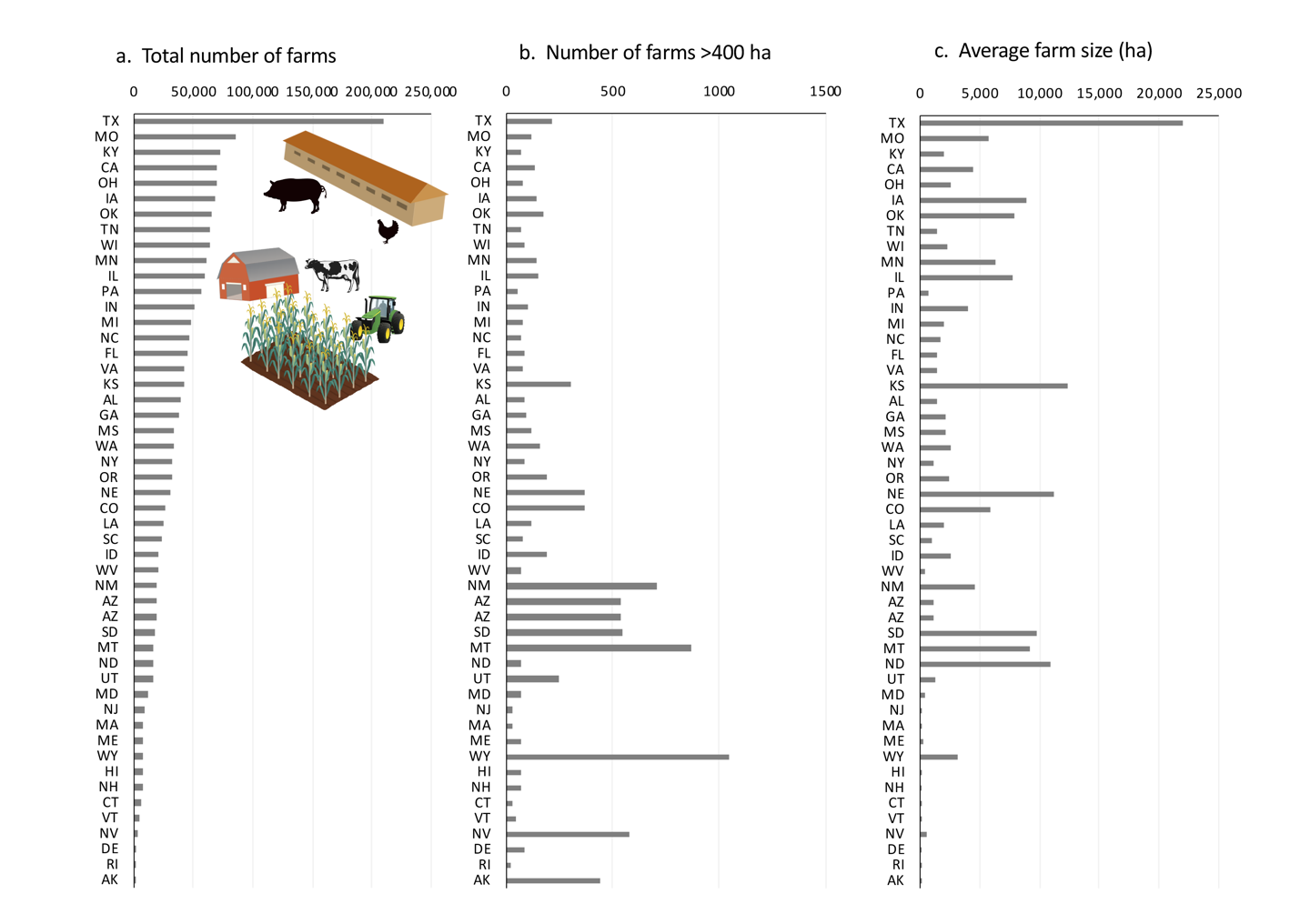


**Online Res. Fig. 2.** Total number of farms (a), farms of a size >400 ha (b) and average farm size (c) by state as of the year 2012. States are ranked based on total farms. Data are from <http://cropinsuranceinamerica.org/wp-content/uploads/NE-State-Fact-Sheet-2015.pdf>. Symbols and icons are from the University of Maryland Center for Environmental Science (UMCES) Integration and Application Network (IAN) or from Vectorstock used under an expanded license.

**Online Res. Fig. 3.** Total commercial fertilizer use as (a) N and (b) P by state as of 2011. Data derived from the US EPA (<https://www.epa.gov/nutrient-policy-data/commercial-fertilizer-purchased>). States are ranked based on total use. Fertilizer icons from UMCES-IAN.

**Online Res. Fig. 4.** Animal inventories by state as of 2019 for (a) cattle, (b) dairy cows, and (c) hogs. Data are from USDA ([www.nass.usda.gov/Statitics_by_State/index.php](http://www.nass.usda.gov/Statitics_by_State/index.php)). States are ranked based on total animals produced (see also Online Res. Fig. 5). Animal icons are from Vectorstock used under an expanded license.

**Online Res. Fig. 5.** As for Online Res. Fig. 4, except for (a) broilers, and (b) turkeys.


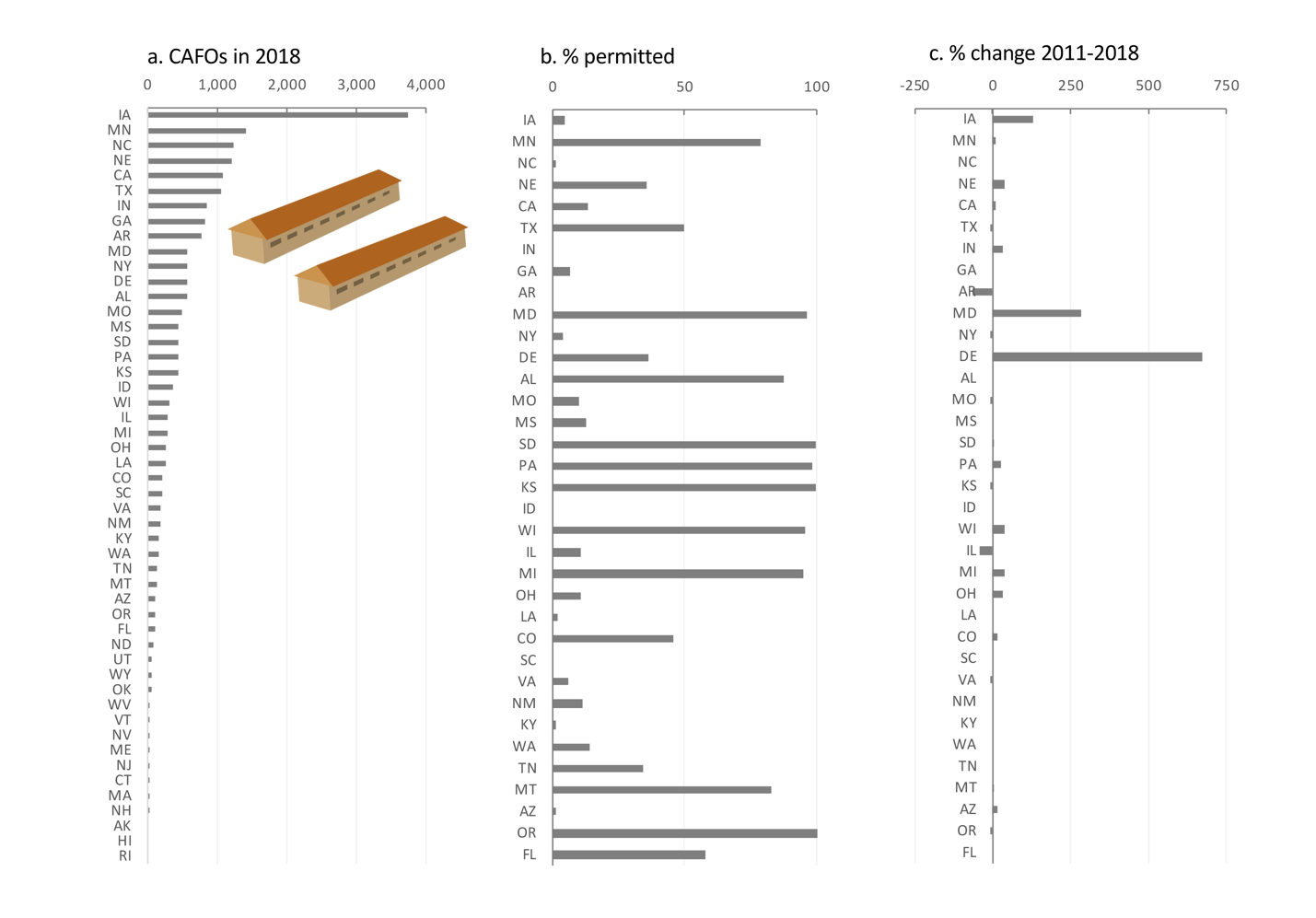


**Online Res. Fig. 6.** Total number of CAFOs by state as of 2018 (a), and of those states with more than 100 CAFOs, the percent of those that are permitted under NPDES of the Clean Water act (b), and the percent change in CAFO numbers from 2011-2018 (c). The inventory for 2018 and percent permitted are derived from the EPA (<https://www.epa.gov/sites/production/files/2019-09/documents/cafo_tracksum_endyear_2018.pdf>), and the percent change over time compares those data with those reported by Walljasper (2018). The CAFO icon is from UMCES-IAN.

**Online Res. Fig. 7**. Panel (a) color key to maps in Online Res. Figs 7-8. Panel (b) cattle inventory for 2012, and (c) for 2017. Figures are from Food and Water Watch (2015, 2020). Animal icon is from Vectorstock used under an expanded license.

**Online Res. Fig. 8.** Inventory for CAFOs for dairy in 1997 (a), 2012 (b), and 2017 (c); for hogs in 1997 (d), 2012 (e) and 2017 (f, with insert map of Iowa); and for broilers (g), 2012 (h) and 2017 (i). Figures are from Food and Water Watch (2015, 2020). Animal icons are from Vectorstock used under an expanded license.

**Online Res. Fig. 9.** NH_3_ emission (as MT for the year 2014) by state from (a) fertilizer, and (b) livestock waste. States are ranked based on total emissions. Data are from the EPA National Emissions Inventory (NEI) data (https://www.epa.gov/air-emissions-inventories/2017-national-emissions-inventory-nei-data).

**Online Res. Fig. 10**. Greenhouse gas emissions as CO_2_ equivalents by state in million MT from (a) enteric fermentation; (b) livestock waste from CH_4_; (c) livestock waste from N_2_O. Data are from USDA (2016) based on the year 2013 (http://dx.doi.org/10.15482/USDA.ADC/1264344).

**Online Res. Fig. 11**. (a) Human population by state as of 2019, (b) N produced and (c) P produced per day in million MT, and (d) estimated wastewater infrastructure investment needed ($/person/yr over next 20 years). Human population data are from [www.worldpopulationreview.com/states/](http://www.worldpopulationreview.com/states/), and wastewater infrastructure need data are from infrastructurereportcard.org. Sewage plant icon is from UMCES-IAN.
